# Canine diffuse large B-cell lymphoma downregulates the activity of CD8 + T-cells through tumor-derived extracellular vesicles

**DOI:** 10.1186/s12935-023-03104-4

**Published:** 2023-10-26

**Authors:** Hsin-Pei Weng, Chiao-Hsu Ke, Chun-Wei Tung, Akiyoshi Tani, Chia-Chi Wang, Wen-Yuan Yang, Yu-Shan Wang, Winston Han, Chi-Hsun Liao, Hirotaka Tomiyasu, Chen-Si Lin

**Affiliations:** 1 Department of Veterinary Medicine, School of Veterinary Medicine, National Taiwan University, No.1 Sec.4 Roosevelt Rd, Taipei, 10617 Taiwan ROC; 2 Institute of Biotechnology and Pharmaceutical Research, National Health Research Institutes, 35053 Miaoli, Taiwan; 3 Graduate Institute of Data Science, College of Management, Taipei Medical University, 106, Taipei, Taiwan; 4 Doctoral Degree Program in Toxicology, College of Pharmacy, Kaohsiung Medical University, 80708 Kaohsiung, Taiwan; 5 Department of Veterinary Internal Medicine, Graduate School of Agricultural and Life Sciences, The University of Tokyo, 1-1-1, Yayoi, Bunkyo-Ku, Tokyo, 113-8657 Japan; 6 Zoonoses Research Center and School of Veterinary Medicine, National Taiwan University, Taipei, 106 Taiwan; 7 Lab. 2612, Rekiin Biotech Inc, Taipei, 114737 Taiwan

**Keywords:** Extracellular vesicles, Diffuse large B-cell lymphoma, Immune suppression, CD8 + T-cell, CTLA-4 blockade

## Abstract

**Background:**

Tumor-derived extracellular vesicles (EVs) have been proposed as the essential mediator between host immunity and cancer development. These EVs conduct cellular communication to facilitate tumor growth, enable invasion and metastasis, and shape the favorable tumor microenvironment. Lymphoma is one of the most common hematological malignancies in humans and dogs. Effective T-cell responses are required for the control of these malignancies. However, the immune crosstalk between CD8 + T-cells, which dominates anti-tumor responses, and canine lymphoma has rarely been described.

**Methods:**

This study investigates the immune manipulating effects of EVs, produced from the clinical cases and cell line of canine B cell lymphoma, on CD8 + T-cells isolated from canine donors.

**Results:**

Lymphoma-derived EVs lead to the apoptosis of CD8 + T-cells. Furthermore, EVs trigger the overexpression of CTLA-4 on CD8 + T-cells, which indicates that EV blockade could serve as a potential therapeutic strategy for lymphoma patients. Notably, EVs transform the CD8 + T-cells into regulatory phenotypes by upregulating their PD-1, PD-L1, and FoxP3 mRNA expression. The regulatory CD8 + T-cells secret the panel of inhibitory cytokines and angiogenic factors and thus create a pro-tumorigenic microenvironment.

**Conclusion:**

In summary, the current study demonstrated that the EVs derived from canine B cell lymphoma impaired the anti-tumor activity of CD8 + T-cells and manipulated the possible induction of regulatory CD8 + T-cells to fail the activation of host cellular immunity.

**Supplementary Information:**

The online version contains supplementary material available at 10.1186/s12935-023-03104-4.

## Background

Small extracellular vesicles (EVs) are cell-secreted substances containing RNA, DNA, and proteins, with average diameters less than 200 nm [1, 2]. All kinds of cells, especially tumor cells, rely on secreting these tiny particles to exert significant physiological effects. Cancer-derived small EVs participate in communication between cells, downregulate the functions of host immunity [3], and thus promote tumor progression [4]. These particles have been disclosed to suppress anti-tumor responses by augmenting the power and lifespan of regulatory T-cells, generating myeloid-derived suppressor cells (MDSC), and blocking the maturation of dendritic cells and macrophages [3, 5–7]. In addition, several mechanisms have also been proposed to illustrate how these small EVs may arrest T-cell functions. EVs block the differentiation of Th1 lymphocytes [8], inhibit NKT cell activity [9], and terminate the anti-tumor effects of CD8 + T-cells [10]. The EVs can suppress CD8 + cytotoxic T-cells (CTLs) by releasing apoptosis-inducing molecules, such as Fas ligand (FasL), PD-L1, and TNF-related apoptosis-inducing ligand (TRAIL) [11, 12]. These apoptotic signals subsequently induce the presence of phosphatidylserine (PS) on the outer plasma membrane, caspase 3 cleavages, and DNA fragmentation [12]. EVs also disturb the activation of CD8 + T-cells. They can suppress TCR-dependent translocation of NFκB and NFAT and downregulate CD69 and CD107a expression, thereby inhibiting T cell proliferation and cytokine production [13]. Therefore, small EVs derived from the tumor cells can effectively enable cancer progression by manipulating the development and activation of CD8 + T-cells.

Lymphoma is one of the most common hematological malignancies in both humans and dogs [14]. In diffuse large B-cell lymphoma (DLBCL), the most common subtype of human B-cell lymphomas, the numbers of T-cells are positively correlated to the prognosis in patients [15]. This highlights the significance of “functional” T-cells in B-cell lymphoma patients. Tumor-induced T-cell fatigue is common in this patient group. Overexpression of LAG-3, a T-cell exhaustion marker, in B-cell lymphoma patients is associated with loss of function in CD8 + T-cells [16]. Furthermore, subjects with human immunodeficiency virus-1 (HIV-1), acquired immune deficiency syndrome (AIDS), and other immunodeficient diseases have elevated rates of B-cell lymphoma [17, 18], which suggest that effective T-cell responses are required for the prevention and control of B-cell malignancies. Although the roles of tumor behaviors in human lymphoma have been well-described [19], the potential mechanisms by which the B-cell lymphoma cells inhibit the T-cells remain unclear. Similarly, in veterinary medicine, research relating to the interaction between canine B-cell lymphoma and CD8 + T-cell functions has been rarely reported. As human and canine lymphoma share high similarities in morphology, tumor genetics, disease progression, and treatment responses [14], this study investigated the interaction between DLBCL and host immunity, focusing on T-cells.

In previous research, we comprehensively analyzed the miRNA and protein profiles of EVs derived from CLBL-1, a DLBCL cell line [2]. The pan-screening results suggested that CLBL-1-derived EVs included several immune regulating components such as CD81 [20], FAS [21], and HSP71 [22], though the crosstalk between host immunity and the EVs is not well elucidated. Furthermore, the effects of lymphoma-derived EVs on CD8 + T-cells remain controversial. Herein, we investigate if EVs collected from canine DLBCL patients and the cell line could lead to the dysfunction of T-cell-mediated anti-tumor immune responses. The influence of EVs on host immune communication in canine CD8 + T-cells is disclosed for the first time in this study.

## Methods

### Cell culture and EVs purification

A canine diffuse large B-cell lymphoma (CLBL-1) [23] cell line was kindly provided by Dr. Barbara C. Rütgen, University of Veterinary Medicine (Vienna, Austria). CLBL-1 cells were maintained in RPMI-1640 (Thermo Fisher Scientific, Waltham, MA, USA) supplemented with 10% fetal bovine serum (Thermo Fisher Scientific) and 1% antibiotic–antimycotic (Thermo Fisher Scientific). The cells were cultured at 37 °C in a humidified atmosphere containing 5% CO_2_. Cells were routinely screened to prevent mycoplasma contamination (Sigma-Aldrich, St. Louis, MO, USA). The EVs were isolated from the culture medium of CLBL-1 as previously described [2]. Briefly, 3 × 10^7^ cells were cultured for 24 h in a serum-free growth medium and separated from the cell culture medium with Total Exosome Isolation Reagent (Thermo Fisher Scientific) according to the manufacturer’s instructions. After the last centrifugation, the EVs were precipitated, followed by the removal of the culture supernatant. The traits of purified EVs, including quantity and average size, were measured using the NanoSight NS300 system (Malvern Instruments, Malvern, UK), which is concordant with the size definition (Additional file 1: Fig. S1) [2]. Isolated EVs were then re-suspended in PBS and lysed with RIPA lysis buffer (Sigma-Aldrich) supplemented with protease inhibitors (Thermo Fisher Scientific) and phosphatase inhibitors (Thermo Fisher Scientific), according to previous study [24]. The concentration of EV protein samples was quantified using Micro BCA Protein Assay (Thermo Fisher Scientific).

### Recruiting canine DLBCL patients and their plasma EV preparations

After the owner's consent, neoplastic lymph nodes were collected from dogs presented to the National Taiwan University Veterinary Hospital (NTUVH). The dogs were diagnosed with DLBCL via flow cytometry using the samples collected with fine-needle aspiration (FNA) of enlarged peripheral lymph nodes (CD21-positive) and further confirmed by histopathology and immunohistochemistry (positive for CD79a). The animal experiment was approved by the Institutional Animal Care and Use Committee, National Taiwan University (IACUC No. NTU-107-EL-00200). Extracellular vesicles (EVs) were isolated from the plasma according to the previous publication [25]. Frozen plasma specimens were thawed and subjected to centrifugation at 3000 × g for 20 min at 4 °C, followed by centrifugation at 12,000 × g for 60 min at 4 °C. The clarified plasma was then ultracentrifuged in an Optima L-100 K ultracentrifuge (Beckman Coulter, Brea, CA, USA) at 100,000 × g for 120 min at 4 °C to pellet the EVs. The supernatant was carefully removed, and the crude EV-containing pellets were resuspended in ice-cold PBS. These resuspended pellets were then floated on a sucrose cushion (30%, D_2_O) (Sigma-Aldrich, Saint Louis, MO, USA) for 60 min at 100,000 × g at 4 °C to remove non-EV protein complexes. After washing, the EVs collected in the sucrose cushion were pelleted for 16 h at 100,000 × g at 10 °C. Finally, the EVs were resuspended in PBS and analyzed using NanoSight NS300 system (Malvern Instruments, Malvern, UK) for nanoparticle tracking analysis.

### Primary culture and purification of canine CD8 + T-cell

Peripheral blood from the dogs was heparinized (10 U/ml) and diluted to twice the original volume with PBS. PBMCs were isolated by Ficoll-Paque density (specific gravity: 1.077) gradient centrifugation (800 g, 35 min, 20 °C) [26]. The signalment, PBMC counts, and comparisons of CD8 + T-cells, by different parameters of healthy dogs in the study, are summarized in Additional file 1: Table S1 and S2. CD8 + T-cells were stimulated as previously described with simple modification [26, 27]. Briefly, PBMCs were stimulated with 2500 IU/mL recombinant human IL-2 (rhIL-2) and 50 µM 2-Mercaptoethanol (Sigma-Aldrich) for 14 days. Phenotypic analysis of lymphocyte subsets (CD4 + and CD8 + cells) was performed using flow cytometric analysis every 3 to 4 days to determine the proportion of CD8 + T-cells (Additional file 1: Fig. S2A). After 14 days, the cultured cells had sufficiently proliferated, and were harvested and processed into purification (Additional file 1: Fig. S2B). The MACS magnetic beads (Miltenyi Biotec Inc., San Diego, CA, USA) with a selection column were utilized according to the instructions with simple modifications [28]. Briefly, the stimulated cells were incubated with mouse anti-CD8 + antibody and secondary goat anti-mouse microbeads (coated with a fluorochrome (FITC)). The incubated cells were positively selected by MACS column (Miltenyi Biotec Inc.) and subsequently washed. Isolation efficiencies were analyzed by flow cytometry (Additional file 1: Fig. S2C). The isolated CD8 + T-cells had a purity of more than 95%, whereas unlabeled cells passed the magnetic field and were located in the flow‐through fraction with a purity of less than 5% (Additional file 1: Fig. S2D).

### Flow cytometry

Fluorochrome-conjugated monoclonal antibodies were used in rat anti-dog CD4-FITC (Bio-Rad, clone: YKIX302.9, Hercules, CA, USA), rat anti-dog CD8 + -FITC (Bio-rad, clone: YCATE55.9), and rat anti-dog CD8 + -PE (Bio-Rad, clone: YCATE55.9). Their corresponding fluorescein-conjugated isotype antibodies were rat IgG2a-FITC (Biolegend, clone: RTK2758, San Diego, CA, USA), rat IgG1-FITC (Bio-Rad), and rat IgG1-PE (Bio-Rad). The mouse anti-human CD152 (CTLA-4)-PE antibody (Ancell Corporation, clone: ANC152.2/8H5, Stillwater, MN, USA), which cross-reacts with canine homologs, and the corresponding isotype antibody, mouse IgG1κ-PE (BD Biosciences, clone: MOPC-21, San Jose, CA, USA), were used. The CD8 + T-cells were harvested, washed twice with PBS, and resuspended in the stain buffer (BD Biosciences). Cells were incubated for 30 min at 4 ℃ before washing and analysis. To measure IFN-*r*, cells were incubated with GolgiPlug (BD Biosciences) at a 1:1500 dilution factor in a complete culture medium for 8 h before washing, fixation, and permeabilization (BD Biosciences). After permeabilization, cells were then stained with mouse anti-bovine IFN-*r*-PE monoclonal antibody (Invitrogen, clone: CC302, Carlsbad, CA, USA), which cross-reacts with canine IFN-r [29]. Samples were analyzed with an LSR Fortessa Flow Cytometer (BD Biosciences). Data were analyzed in FlowJo v10 software (BD Biosciences).

For annexin V and PI staining, the flow cytometric data were obtained by APC Annexin V Apoptosis Detection Kit with PI (Biolegend) according to the manufacturer's instructions. The cell pellets were harvested, washed twice with cell staining buffer, and then double-stained with Annexin V and PI for 15 min at room temperature. The stained cells were analyzed using an LSR Fortessa Flow Cytometer (BD Biosciences) for Annexin V-APC and PI, respectively.

### EV treatment for canine CD8 + T-cells

Isolated CD8 + T-cells were seeded in 24-well plates at a density of 600,000 cells/well with or without EVs. The CD8 + T-cells were maintained in RPMI-1640 supplemented with 10% fetal bovine serum and 1% antibiotic–antimycotic in a humidified atmosphere containing 5% CO_2_ at 37 °C. EVs were added to the CD8 + T-cells at a final concentration of 50, 100, or 200 µg/ml, and the same volume of PBS without EVs was added to the control group. After appropriate incubation, the cells were harvested for further analysis.

### Cell viability

The effects of EVs on CD8 + T-cells were determined by the WST-1 assay (TaKaRa Bio., Japan). CD8 + T-cells were seeded at a density of 50,000 cells/well with 1% serum culture medium in a 96-well flat-bottom plate and treated (100 or 200 µg/ml) with or without (PBS as vehicle control) EVs for 48 and 72 h. After incubation, 10 µl (1:10 in volume) of WST-1 reagent was added to the medium, and the cells were incubated for additional 4 h. The cell viability was determined by measuring the absorbance at 450 and 690 nm with a microplate reader (SpectraMax® M5 Microplate Reader, San Jose, CA, USA).

### RNA isolation, cDNA synthesis, and quantitative PCR

Total RNA was extracted using TRIzol reagent (Invitrogen) according to the manufacturer’s instructions. Briefly, CD8 + T-cells were harvested, washed twice with PBS, resuspended in TRIzol reagent by vortex, and incubated on ice for 10 min. Following chloroform extraction, RNA was precipitated with cold isopropanol. After centrifugation at 10,000 g for 15 min, the supernatant was discharged, and the precipitated pellet was washed with 70% ethanol, dried in a vacuum chamber, and resuspended in diethylpyrocarbonate-treated water (DEPC-water). The RNA concentration was measured with a Nano photometer™ (Implen GmbH, Munich, Germany). Then a total of 1 µg RNA combined with 1 µl of 50 µM Oligo dT primers (Sigma-Aldrich), 1 µl of 50 nM random primers (Sigma-Aldrich), 1 µl of dNTP, and a volume Ulter Pure DEPC Water (Protech, Taipei, Taiwan) were added, followed by incubation at 65 °C for 5 min. The denatured RNA was then chilled on ice for 10 min, following which 4 µl of 5 × first strand buffer (Invitrogen), 2 µl of 100 mM DTT (Invitrogen), 1 µl of RNase-free water, and 1 µl of SuperScript II RT (Invitrogen) were added to the RNA solution.

Quantitative PCR was performed (in triplicate) using SYBR Green PCR Master Mix (Bio-Rad) according to the manufacturer’s instructions in a qPCR machine (Bio-Rad). The primer sequences are listed in Additional file 1: Table S3 according to the NCBI reference sequence and a previous study [30]. The relative amounts of mRNA in each sample were calculated based on its threshold cycle compared with the threshold cycle of the housekeeping gene, OAZ-1. The results were calculated with the following formula: Ct value of relevant gene – Ct value of OAZ-1.

### Cytokine array and cytokine ELISA

Culture supernatants were collected at 24 h from duplicate wells seeded at 50,000 cells/well in a 24-well plate, with 0.5 mL total medium volume per well. Controls were prepared from the wells receiving complete medium only, without EVs. RayBio^®^ C-Series Canine Cytokine Array Kit 1, which can detect a total of 40 individual canine cytokines, was purchased from RayBiotech (RayBiotech, Peachtree Corners, GA, USA) and utilized according to manufacturer instructions. Briefly, after blocking, samples were placed in each well overnight at 4℃. The next day, samples were twice washed, and HRP-streptavidin incubation was performed overnight at 4℃ before a third wash. The next day, chemiluminescence detection was used to recognize the antibody levels expressed in the membrane. For comparison of their expression levels, internal positive controls were used to define the signal strength. Two duplicate spots were used for all samples. Data were analyzed in Microsoft® Excel-based Analysis Software Tools (RayBiotech) for each array kit via automatic analysis. The canine IFN-*r* (R&D Systems), IL-21, TNF-$\mathrm{\alpha },$ and Rantes quantitative ELISA kits (RayBiotech) were applied to analyze these concentrations in the supernatant under the exposure with or w/o EVs.

### Next-generation sequencing and bioinformatic analysis

The purity and quantification of RNA samples were measured using SimpliNano™—Biochrom Spectrophotometers (Biochrom, Holliston, MA, USA). The degradation and integrity of RNA samples were monitored by Qsep 100 DNA/RNA Analyzer (BiOptic Inc., Taipei, Taiwan). For RNA sample preparation, 1 μL total RNA per sample was used as input material. Sequencing libraries were conducted using the KAPA mRNA HyperPrep Kit (KAPA Biosystems, Basel, Switzerland) according to the manufacturer’s instructions, and index codes were added to attribute sequences to all samples. KAPA HiFi HotStart ReadyMix (KAPA Biosystems) was used to amplify the library such that appropriate adapter sequences were at both ends with library amplification primers. Finally, the KAPA Pure Beads system (KAPA Biosystems) was used to purify PCR products, and the library quality was estimated with a Qsep 100 DNA/RNA Analyzer (BiOptic Inc).

Via high-throughput sequencing (Illumina NovaSeq 6000 platform), the original data were collected through the CASAVA base, altered into raw sequenced reads, and saved in FASTQ format. FastQC and MultiQC [31] were used to verify the quality of the fastq files. The received raw paired-end reads were screened by Trimmomatic (v0.38) [32] to remove low-quality reads, trim adaptor sequences, and extirpate poor-quality bases with the following parameters: LEADING:3 TRAILING:3 SLIDINGWINDOW:4:15 MINLEN:30. The high-quality clean reads data were employed in the subsequent analysis. HISAT2 software (v2.1.0) was used to align the reading pair of each sample with the reference genome (e.g., H. sapiens, GRCh38) [33, 34]. Then FeatureCounts (v1.6.0) was employed to calculate the number of reads mapped to individual genes [35]. For gene expression, DEGseq (v1.36.1) [36] was used to normalize the “Trimmed Mean of M-values” (TMM). Based on the negative binomial distribution and Poisson distribution models [37–39], differentially expressed genes (DEGs) were analyzed in R utilizing DEGseq, and the obtained p-value was adjusted using Benjamini and Hochberg's method of controlling FDR. The cluster profiler (v3.10.1) was used to enrich the GO and KEGG pathway analysis of DEGs [40–42].

A gene interaction network of DEGs was established using the Search Tool for the Retrieval of Interacting Genes/Proteins (STRING) network [43] (https://string-db.org/, accessed on 10 Sep 2023) and further analyzed in Cytoscape v.3.8.2 [44]. Then, a Cytoscape plugin, Molecular Complex Detection (MCODE) was employed to obtain the most important cluster of genes in the network. The hub genes were then filtered using the cytoHubba plugin with three different topological analysis methods to avoid bias [45].

### Statistical analysis

The data are described as mean ± standard error of the mean (SEM). Statistical analyses were performed in GraphPad Prism v8. To determine significant differences, the Mann–Whitney U test or two-way ANOVA with Tukey multiple comparisons were utilized. Differences were considered statistically significant at a *p* value of less than 0.05.

## Result

### CD3 + CD8 + T cells are significantly decreased in DLBCL-bearing dogs

**Fig. 1:**
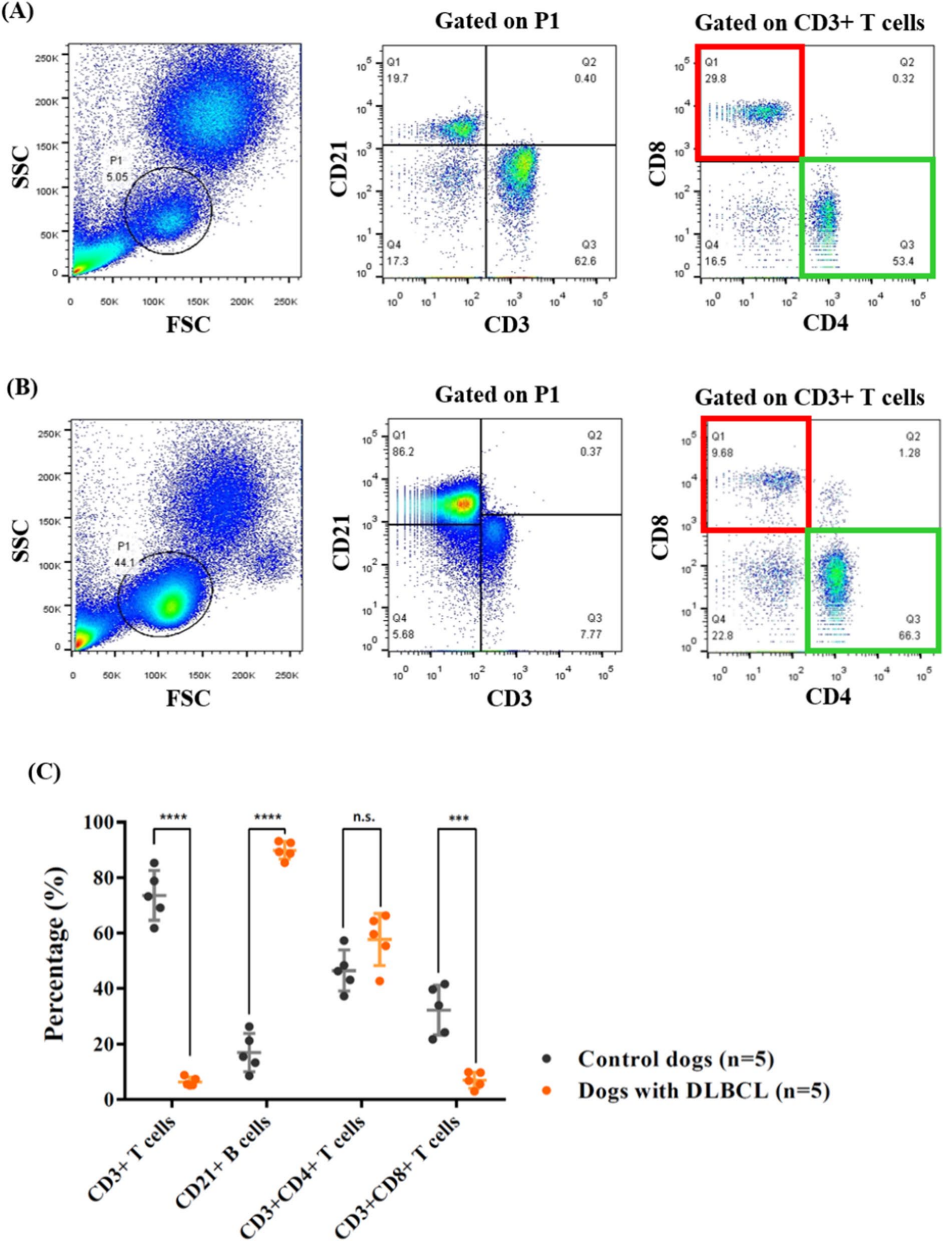
Significant decrease in CD3 + CD8 + double-positive T cells in dogs with DLBCL. **A** Representative flow cytometric figures of PBMCs isolated from healthy dogs and **B** dogs with DLBCL. **C** Statistical analysis of B and T cell subtypes proportions in dogs with or without DLBCL. Bar graphs reflect mean ± SD (n = 5) analyzed by the unpaired Student t-test. *FSC* forward scatter, *SSC* side scatter, *DLBCL* diffuse large B-cell lymphoma, *PBMCs* peripheral blood mononuclear cells; ***,* P* < 0.001; ****, *P* < 0.0001; n.s., no significant difference

CD3 + CD8 + T cells are vital for ant-tumor immunity in many cancer types, including DLBCL; however, cellular immunity is usually severely dampened during aggressive tumor development. We have compared the population of B and T lymphocytes in PBMCs derived from healthy and DLBLC dogs. The results showed that B lymphocytes dominated the PBMCs in B cell lymphoma patients (89.8 $\pm$ 3.1%) (*P* < 0.0001), which was the typical blood profiling in DLBCL patients. Moreover, though both healthy and cancer dogs have similar ratios of CD4 + / CD3 + T cells (46.5 $\pm$ 7.4% v.s. 57.7 $\pm$ 9.4%), DLBCL patients had significantly decreased CD3 + CD8 + T cells (6.9 $\pm$ 3.0%) compared to the healthy dogs (*P* < 0.001) (Fig. 1). These results indicated that reduced CD3 + CD8 + T cells are commonly identified in DLBCL patients.

### CLBL-1-derived EVs induced late apoptosis in CD8 + T-cell

**Fig. 2:**
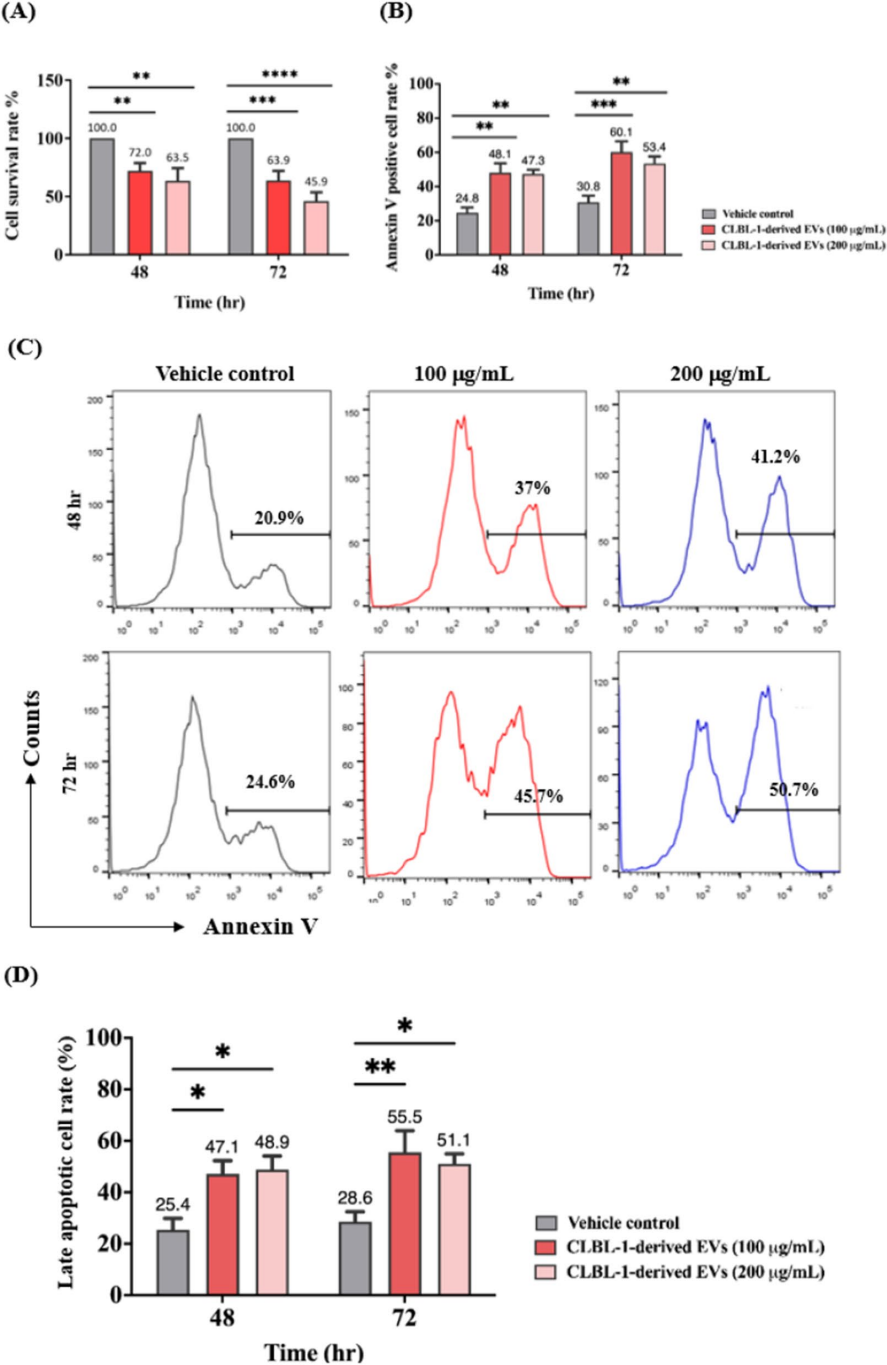

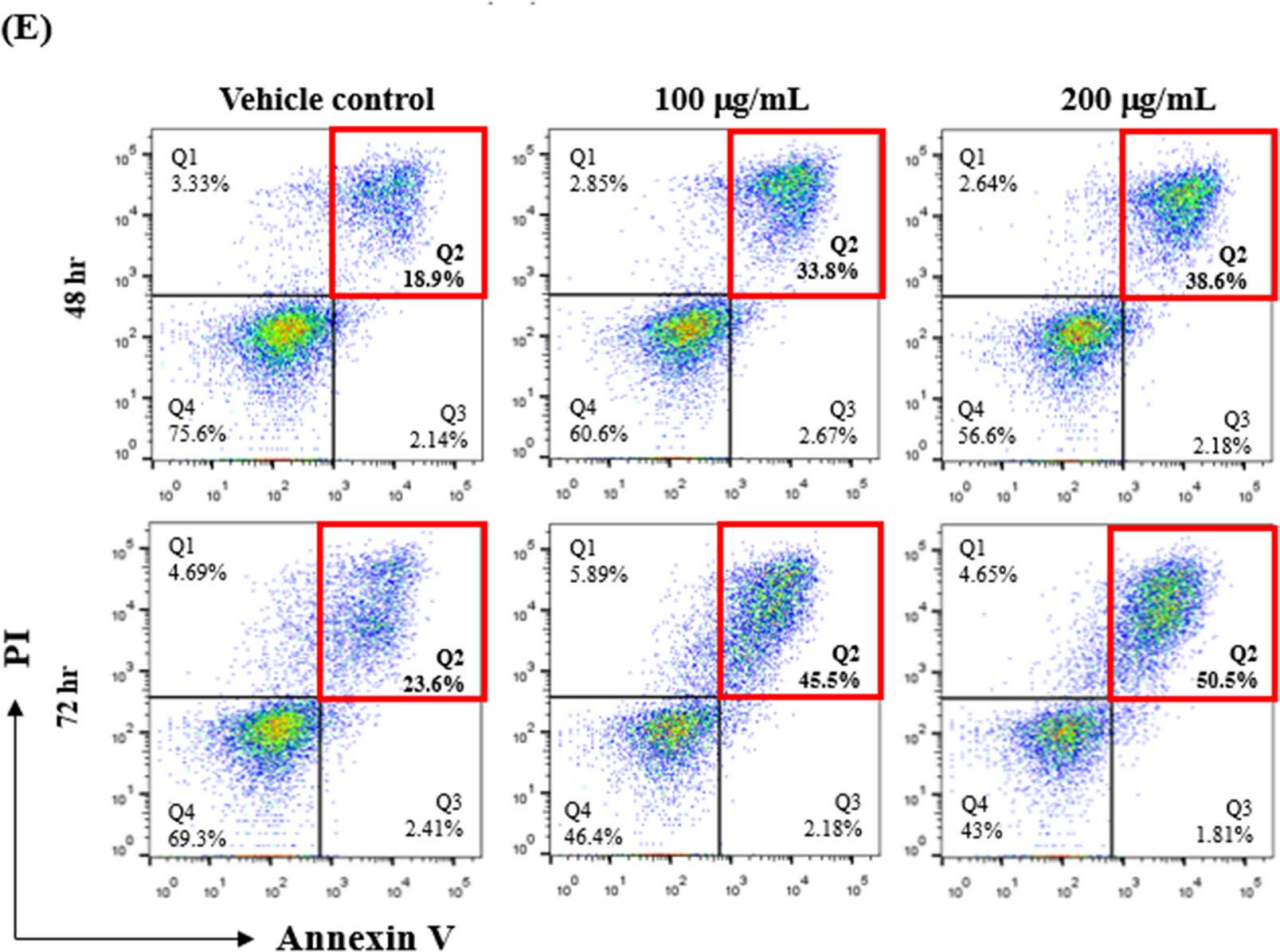
Tumor-derived EVs reduce cell viability and induce late apoptosis in CD8 + T cells. **A** CD8 + T cells were treated with 100 and 200 µg/ml CLBL-1-derived EVs for 48 and 72 h, and WST-1 assay was used to determine cell viability. **B** Quantitative analysis of the ratios of the apoptotic CD8 + T cells (Annexin V +). **C** Representative figures showed each group's flow cytometric analysis of CLBL-derived EV-induced CD8 + T cell apoptosis. **D** Statistical analysis and **E** representative dot-plot of late apoptotic cells by flow cytometry. Numbers represent the ratios of cells in each quadrant. Propidium iodide (PI) fluorescence is measured on the Y-axis, and Annexin V-APC is measured on the X-axis. Data are representative of 3–4 independent experiments. Bar graphs reflect mean ± SEM (n = 5–6) analyzed by two-way ANOVA with Tukey’s post hoc test. **, *P* < 0.01; ***,* P* < 0.001; ****, *P* < 0.0001

The above data demonstrated the decrease of CD8 + T cells in DLBCL dogs; the possibly plummeting mechanisms were first investigated using in vitro model. We prepared EVs from CLBL-1 cells and detected the cytotoxicity of EVs by WST-1 assay. Compared with the vehicle control, EVs (100 µg/mL) reduced the viability of CD8 + T-cells to 72.0% ± 6.9% and 63.9% ± 8.1% at 48 and 72 h, respectively. Increasing the EV dosage (200 µg/mL) further decreased CD8 + T-cell viability, with remaining survival rates of 63.5% ± 11.0% and 45.9% ± 7.7% after 48 and 72 h incubation (Fig. 2A). Annexin V/PI staining results indicated that apoptosis played the significant role in reducing T-cell viability by CLBL-1-derived EVs. A total of 48.1% ± 5.4% and 47.3% ± 2.5% of CD8 + T-cells were Annexin V + after 100 and 200 µg/mL EV treatment for 48 h, and 60.1% ± 6.4% and 53.4% ± 4.2% of apoptotic cells were found after 72 h of incubation (Fig. 2B and C). The EV-induced late apoptosis (Annexin V + /PI +) was also higher than that in the control group, in which 47.1% ± 5.2% and 48.9% ± 5.2% of cells were late apoptosis after 48 h incubation, and 55.5% ± 8.4% and 51.1% ± 3.8% of late dead cells were found for the 72 h EV treatment (Fig. 2D and E). Together, these results indicated that CLBL-1-derived EVs could decrease cell viability by triggering apoptosis.

### CLBL-1-derived EVs disturbed IFN-r-secreting CD8 + T-cell

**Fig. 3:**
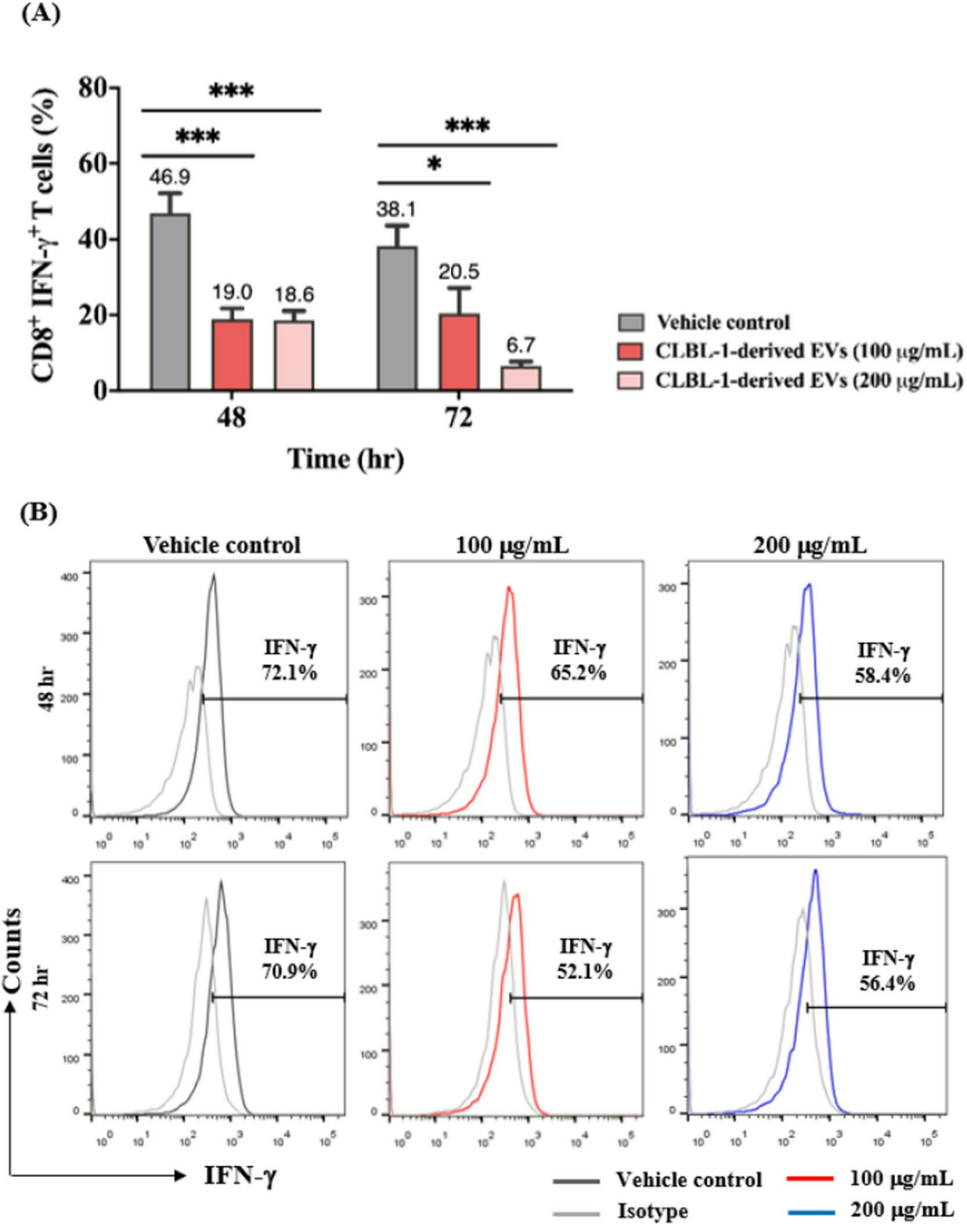

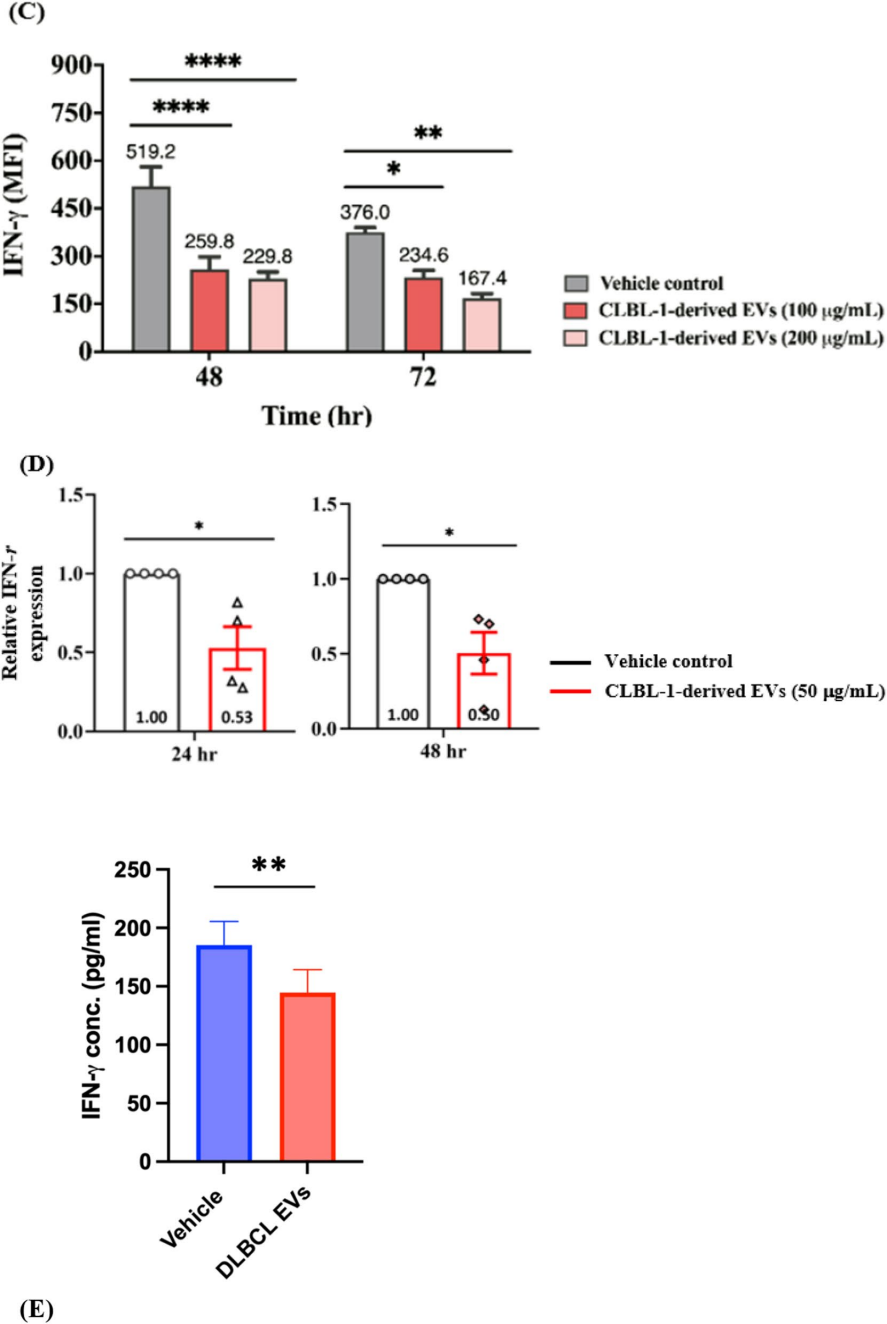
CD8 + T cells exhibit debilitated IFN-r secretion ability after tumor-derived EV incubation. **A** IFN-r expression in CD8 + T cells after incubation of EVs. **B** Representative flow cytometric figures of IFN-*r* secretion (gated on CD8 + T cells) with EVs treated and **C** corresponding quantification of mean fluorescence intensity (MFI). **D** Relative IFN-*r* gene expression of CD8 + T cells evaluated by quantitative PCR at 24 and 48 h. IFN-*r* genes were normalized to control. Data are representative of 3–4 independent experiments. **E** IFN-*r* concentration in the supernatant collected from CD8 + T cells treated with or w/o EVs for 48 h. Bar graphs reflect mean ± SEM (n = 5–6) analyzed by two-way ANOVA with Tukey's post hoc, Mann–Whitney U test (quantitative PCR) or Student’s t-test (IFN-*r* ELISA). *, *P* < 0.05; **, *P* < 0.01; ***,* P* < 0.001; ****, *P* < 0.0001

A decrease in viability and induction of late apoptosis in CD8 + T-cells after EV treatment was found (Fig. 2). These findings indicated that tumor-derived EVs could influence immune functions by decreasing the amounts of CD8 + T-cells. In addition, we further speculated that the EV incubation also altered the immune functions or regulatory molecules. To clarify the hypothesis, we then assessed IFN-*r*, which plays a vital role in the immune abilities of T-cells, on EV-treated CD8 + T-cells. CD8 + /IFN-*r* + T-cells were significantly diminished after the EV treatment (Fig. 3A). The total of 46.9% ± 5.3% CD8 + /IFN-*r* + T-cells decreased to 19.0% ± 2.8% (100 µg/mL) and 18.6% ± 2.5% (200 µg/mL) after 48 h incubation. Concordantly, only 20.5% ± 6.7% (100 µg/mL) and 6.7% ± 1.1% (200 µg/mL) CD8 + /IFN-*r* + T-cells remained after the 72 h incubation compared with the control group (38.1% ± 5.5%). As shown in Fig. 3B, IFN-*r*-producing CD8 + T-cells significantly decreased, as did the corresponding mean fluorescence intensity (MFI) (Fig. 3C).

To gain more insight into the potential effects underlying immune regulation in CD8 + T-cells, we examined the mRNA expression of IFN-*r* in CD8 + T-cells with or without EV incubation. The mean IFN-*r* transcript expression was significantly reduced to 0.53- and 0.50-fold-change compared with the vehicle control after 24 and 48 h EV incubations (50 µg/mL) (Fig. 3D). Similar findings could be found in the culture media of CD8 + T cells with and w/o EV incubation, the IFN-*r* concentration was decreased when exposing CLBL-1-derived EVs (Fig. 3E). These results indicated that tumor-derived EVs not only triggered late apoptosis but also weakened the IFN-*r* secretion of CD8 + T-cells. These findings enabled us to investigate whether other immune-regulatory impacts were caused by the EVs on CD8 + T-cells.

### CTLA-4 molecule and transcript levels were elevated in CD8 + T-cells treated by the CLBL-1-derived extracellular vesicles

**Fig. 4:**
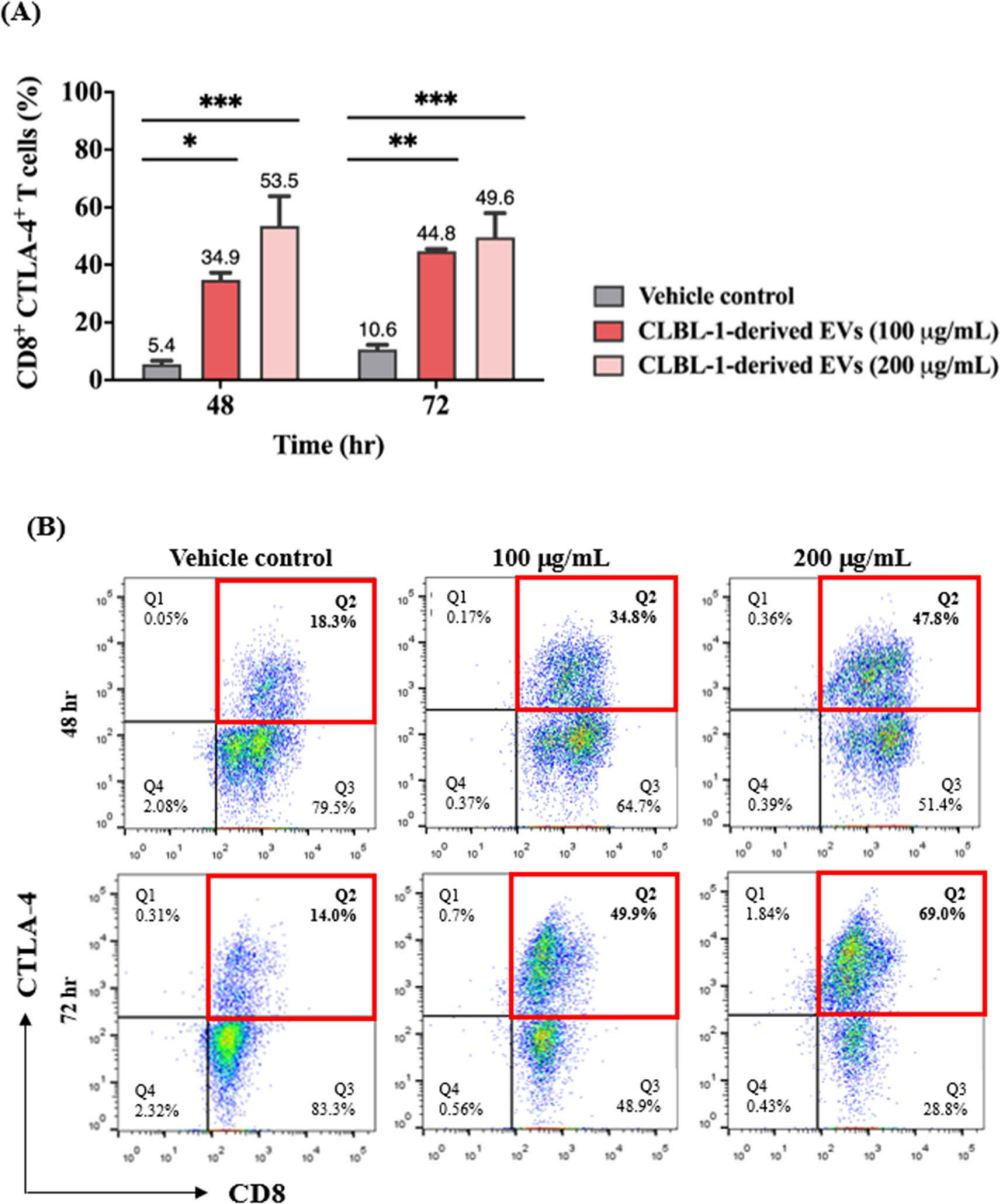

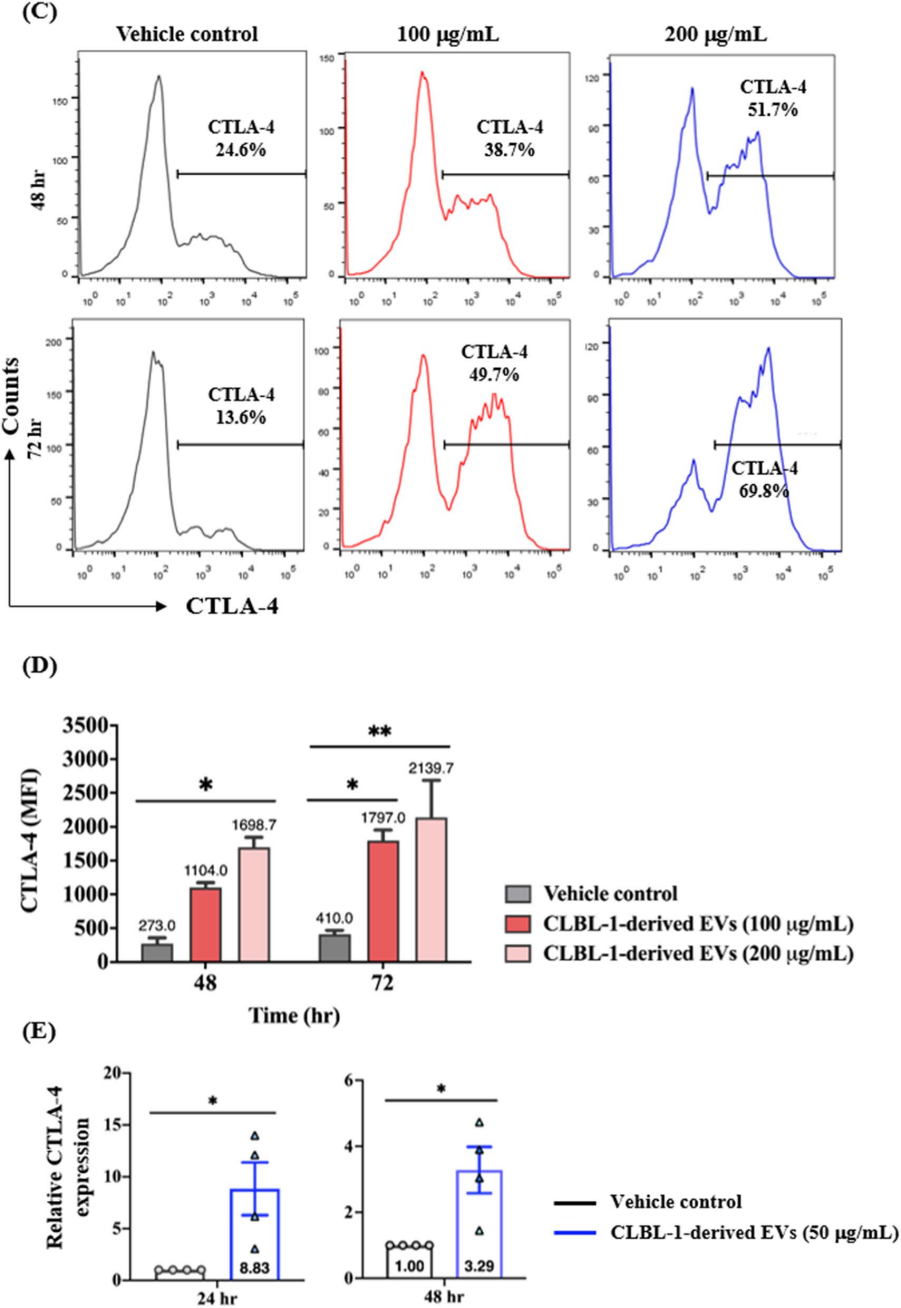
Tumor-derived EVs augmented the overexpression of CTLA-4 in CD8 + T cells. **A** Percentage and **B** representative figures of CTLA-4-expressing CD8 + T cells after incubation of EVs. **C** Representative flow cytometric figures of CTLA-4 expression (gated on CD8 + T cells) with EVs treated and **D** corresponding quantification of mean fluorescence intensity (MFI). **E** CTLA-4 gene expression of CD8 + T cells evaluated by quantitative PCR at 24 and 48 h. CTLA-4 genes were normalized to control. Data are representative of 3–4 independent experiments. Bar graphs reflect mean ± SEM (n = 5–6) analyzed by two-way ANOVA with Tukey’s post hoc test or Mann–Whitney U test (quantitative PCR). *, *P* < 0.05; **, *P* < 0.01; ***,* P* < 0.001; ****, *P* < 0.0001

The aforementioned findings suggested that the EV treatment resulted in a significantly lower frequency of CD8 + /IFN-*r* + T-cells. We next evaluated the expression of CTLA-4, a well-known inhibitory molecule, on CD8 + T-cells. After EV incubation, the CD8 + /CTLA-4 + T-cells significantly increased, indicating that the EV possibly caused the immune regulatory function of the CD8 + T-cells. As shown in Fig. 4A and its corresponding representative figures (Fig. 4B), CD8 + /CTLA-4 + T-cells were outnumbered by 34.9% ± 2.4% (100 µg/mL) and 53.5% ± 10.3% (200 µg/mL) compared with the control group (5.4% ± 1.2%) after 48 h incubation. With a longer period (72 h), similarly, a total of 10.6% ± 1.6% CD8 + /CTLA-4 + T-cells significantly increased to 44.8% ± 0.7% (100 µg/mL) and 49.6% ± 8.4% (200 µg/mL). Furthermore, heightened CTLA-4-expressing CD8 + T-cells were found after EV incubation (Fig. 4C), and corresponding MFI also increased (Fig. 4D).

The transcript levels of CTLA-4 were evaluated as well. The mean relative CTLA-4 expression was significantly enhanced by 8.83-fold and 3.29-fold change compared with vehicle control after 24 and 48 h treatment (50 µg/mL) (Fig. 4E). Together, these results suggested that EV treatment effectively changed the immune behaviors in CD8 + T-cells by up-regulating the CTLA-4 molecule.

### The inhibitory microenvironment sculpted by the CLBL-1-derived EVs contributes to the generation of regulatory CD8 + T-cells

**Fig. 5:**
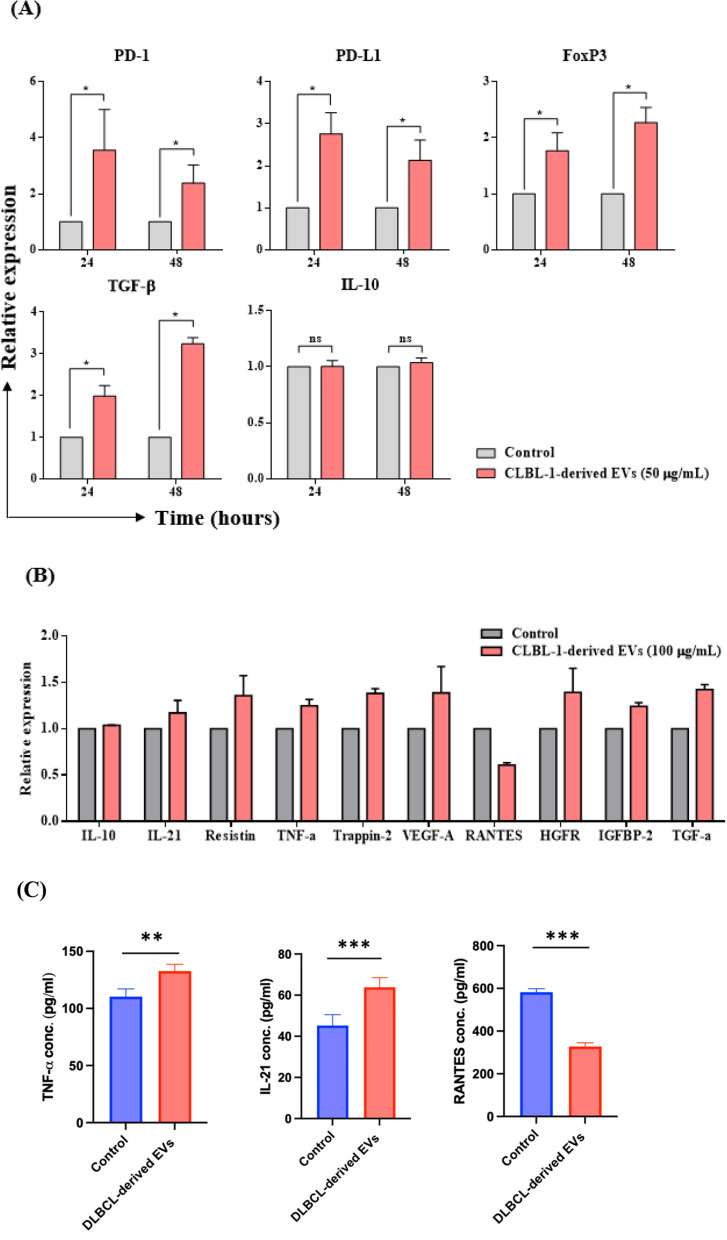
Quantifying inhibitory gene expression and the cytokine secretory profiles in the culture medium of the CD8 + T cells. **A** Gene expression of CD8 + T cells was evaluated by quantitative PCR at 24 and 48 h. Target genes were normalized to control. Data are representative of 3–4 independent experiments. **B** Relative expression of the cytokines between control and the CLBL-1-derived EV-treated CD8 + T cells (n = 2). **C** IL-21, TNF-$\alpha$, and RANTES concentrations in the supernatant collected from CD8 + T cells treated with or w/o EVs for 24 h. Bar graphs reflect mean ± SEM and were analyzed by Mann–Whitney U test or Student’s t-test (ELISA). *, *P* < 0.05; **, *P* < 0.01; ***,* P* < 0.001

EV treatment induced the overexpression of CTLA-4, the receptor known to usually co-work with PD-1 and negatively regulate T-cell functions. Therefore, we further probed how CLBL-1-derived EVs (50 µg/mL) managed the immune regulatory microenvironment. The mRNA levels of PD-1 (Mean, 3.56-fold and 2.38-fold change in 24 and 48 h) and PD-L1 (Mean, 2.76-fold and 2.13-fold change in 24 and 48 h) were significantly increased in CD8 + T-cells. Transforming growth factor-β (TGF-β), which is released by apoptotic T-cells [46] and has been described as playing a role in the suppressive activities of regulatory CD8 + T-cells [47], was elevated in EV-treated T-cells (Mean, 1.99-fold and 3.24-fold change in 24 and 48 h). Furthermore, the key regulatory T-cell transcript factor, FoxP3 [48–50], which can correlate with the suppressive potential of human CD8 + T regs [47], also significantly increased (Mean, 1.77-fold and 2.27-fold change in 24 and 48 h) (Fig. 5A). These findings suggested that the CD8 + T-cell might transform into a regulatory phenotype under the impact of EVs.

The cytokine array (Additional file 1: Fig. S3) was employed to determine the different tumor microenvironments created by the regulatory CD8 + T-cells (under the impact of EVs) compared with their regular counterparts. The differentially expressed cytokines fell into three broad categories. First, the immune suppressive cytokines within the tumor microenvironments, including IL-10 [51] and IL-21 [52], were predominant. Second, the factors that could inhibit the functions of cytotoxic T-cells or enhance the expansion of regulatory T-cells, such as Resistin [53], tumor necrosis factor α (TNF-α) [54], and Trappin-2, were significantly increased [55]. Rantes (CCL5) [56], which can induce anti-tumor immunity and cooperate with Th1 cytokines, such as IL-2 and IFN-γ, was found to be decreased after EV treatment on CD8 + T-cells. Third, angiogenesis-related cytokines were increased, including vascular endothelial growth factor A (VEGF-A), hepatocyte growth factor receptor (HGFR) [57], insulin-like growth factor-binding protein 2 (IGBP-2) [58], and transforming growth factor α (TGF-α) [59] (Fig. 5B). These results indicated that tumor-derived EVs diminished the normal functions of CD8 + T-cells by inducing the regulatory molecules and cytokines. Moreover, these EV-treated T-cells also produced angiogenic factors with pro-tumorigenic effects.

### The differentially expressed genes involved in the regulation and proliferation of CD8 + T-cells

**Fig. 6:**
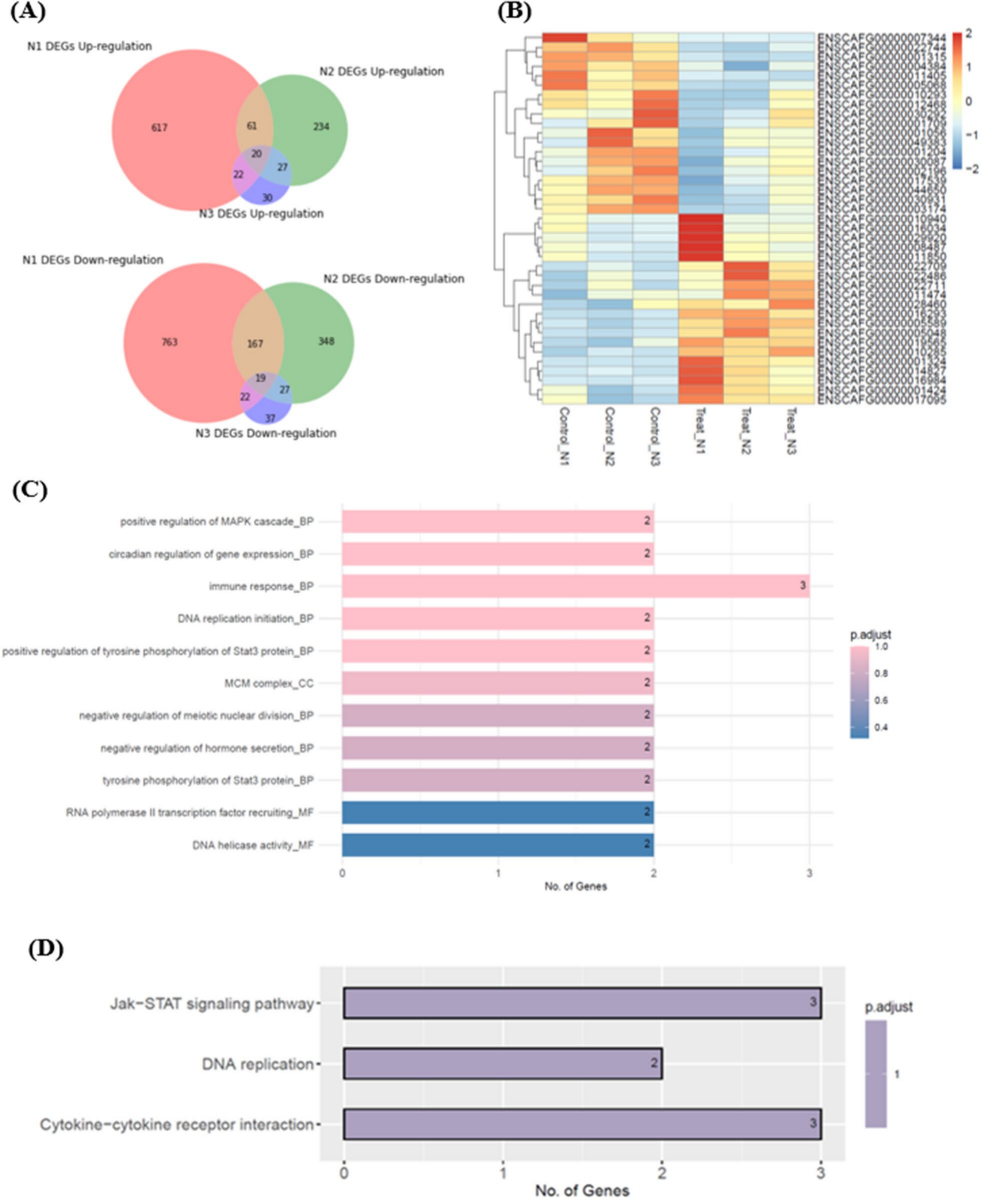
Gene expression profiles of DEGs and enriched GO and KEGG pathways the DEGs involved. **A** The Venn Diagram showed the overlapping upregulated (upper figure) and downregulated (lower figure) DEGs among triplicate experiments. **B** Heatmap of gene expression profiles between CD8 + T cells and EV-treated-CD8 + T cells by hierarchical clustering. The gene abundance is shown with color from high (red) to low (blue). **C** Representative enriched GO term, including BP, CC, and MF. **D** Enriched KEGG pathways were performed for the DEGs. The color bar represents adjusted *P* value, and the numbers in the bars give the gene numbers involved in the certain signal pathway. *DEGs* differentially expressed genes, *GO* Gene Ontology, *KEGG* Kyoto Encyclopedia of Genes and Genomes, *BP* biological process, *CC* cellular component, *MF* molecular function

EV produced from B-cell lymphoma has been proven to significantly reduce cell viability, inhibit IFN-*r* secretion, and upregulate immune-suppressive molecules of CD8 + T-cells. To further probe the potential mechanisms of EVs on immune regulation, gene expression analysis of CD8 + T-cells, with or without EV treatment, was conducted by RNA sequencing. In sample one, 1380 differentially expressed genes (DEGs) were identified, containing 617 upregulated and 763 down-regulated genes. In sample two, 582 genes were filtered as DEGs, 234 genes were upregulated, and 348 genes were defined as down-regulated genes. A total of 67 DEGs were identified in the third sample, including 30 upregulated and 37 down-regulated genes (Fig. 6A). With a *p*-value < 0.05, 39 overlapping DEGs from the three samples were identified (Additional file 1: Table S4 and S5). The heatmap showed the DEG pattern changes in CD8 + T-cells with or w/o EV treatment, which correlated with the dysfunction of EV-treated CD8 + T cells and revealed the potentially deactivated T-cell mechanisms (Fig. 6B). Functional analyses, including GO terms and KEGG pathways for DEGs, were conducted. Most of the enriched GO terms were related to the unbalance of cell proliferation and immune regulation, including the decreased ‘positive regulation of MAPK cascade,’ ‘DNA replication initiation,’ and ‘tyrosine phosphorylation of Stat3 protein.’ (Fig. 6C). Enriched KEGG pathways were related to the downregulation of ‘JAK-STAT signaling pathway,’ ‘DNA replication,’ and some growth-related processes (Fig. 6D). These results disclosed the pieces of evidence that EV-treated CD8 + T-cells possessed poor growth ability and decreased immune functions.

**Fig. 7:**
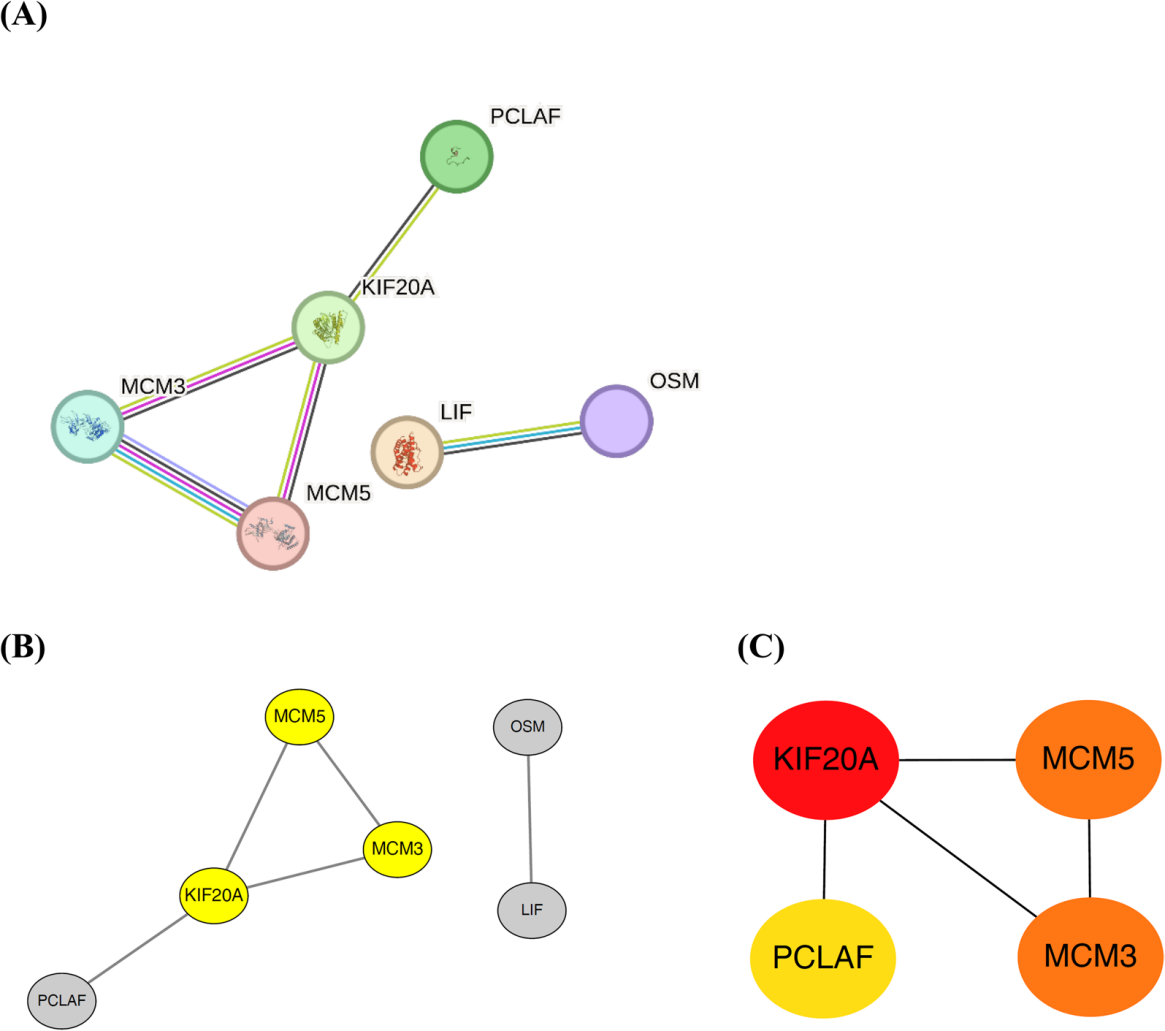
Identification of hub genes from the DEGs. **A** Subnet and clustering analysis of the network. **B** The most import module (node numbers: 3) was extracted and labeled in yellow via Cytoscape using the MCODE plugin **C** The hub genes were identified by MCC topological CytoHubba methods. Red, orange, and yellow circles represent the top one, two, and three important genes. Lines indicate the interactions between identified genes. *DEGs* differentially expressed genes, *MCC* maximal clique centrality

To further elucidate the interaction between the identified DEGs, the interaction network was constructed using the STRING database. Among 39 DEGs, two clusters including six genes were identified as the most correlated genes (Fig. 7A). The most vital module was analyzed using the Cytoscape MCODE plugin. As shown in Fig. 7B, the module, including *KIF20A*, *MCM3*, and *MCM5*, was identified. To select the top 10% of connectivity from all DEGs, the hub genes were identified with three topological analysis methods, maximal clique centrality (MCC), maximum neighborhood component (MNC), and density of maximum neighborhood component (DMNC). Notably, when comparing the genes obtained from these three methods, the results were consistent. The top four hub genes were *KIF20A*, *MCM3*, *MCM5*, and *PCLAF*, respectively (Fig. 7C, only MCC results were shown). Interestingly, *KIF20A* is a well-known tumor antigen [60], and *MCM3* and *MCM5* [61] are critical controllers for regulating cell cycles. Taken together, the above data imply that the breakdown of CD8 + T-cell functionality is attributed to a cascade of signaling axes related to T-cell activation and proliferation."

### EVs extracted from the plasma of clinical DLBCL patients significantly inhibited the activation of CD8 + T cells

**Fig. 8:**
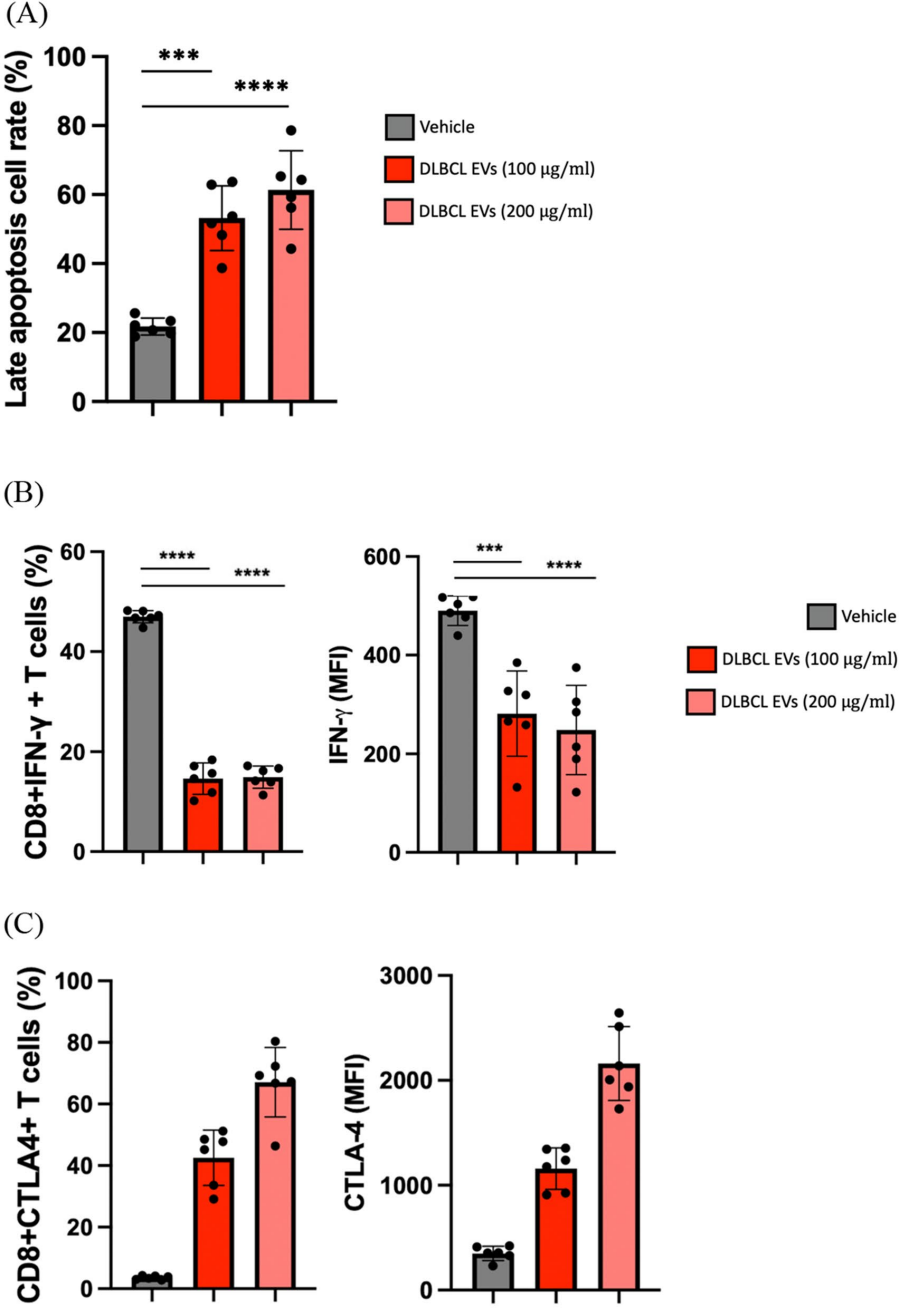

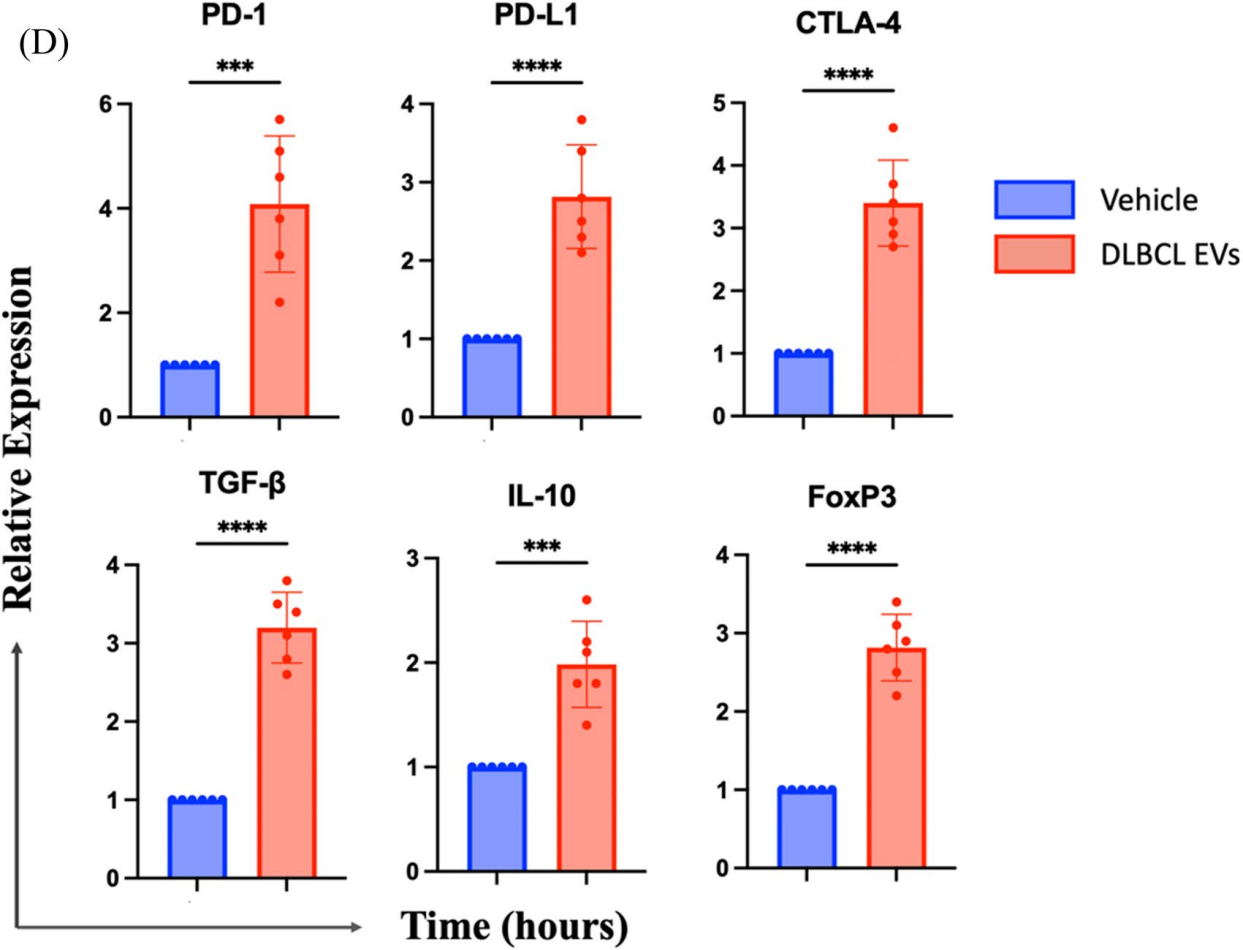
EVs prepared from DLBCL dogs diminished the anti-tumor activity of CD8 + T cells. **A** CD8 + T cells were co-incubated with 100 and 200 µg/ml DLBCL-derived EVs for 48 h, and the apoptotic rate (Annexin V + /PI +) was analyzed by flow cytometry. **B** Percentage and corresponding quantification of mean fluorescence intensity (MFI) of IFN-r; **C** CLTA-4 expression in CD8 + T cells after incubation of DLBCL EVs. **D** Gene expression of immune-regulatory molecules was detected in CD8 + T cells treated with DLBCL EVs at 24 h. Data are obtained from the EVs purified from the blood of 6 DLBCL dogs. Bar graphs reflect mean ± SEM (n = 6) analyzed by two-way ANOVA with Tukey's post hoc or Mann–Whitney U test (quantitative PCR). ***,* P* < 0.001; ****, *P* < 0.0001

To further validate the immunosuppressive effect of B-cell lymphoma on CD8 + T cells, we isolated extracellular vesicles (EVs) from the blood of patients clinically diagnosed with Diffuse Large B-cell Lymphoma to investigate their impact on T cells. A total of six canines diagnosed with DLBCL were enrolled in the study (Table 1). The results displayed that, similar to our previous findings in CLBL-1, the clinical DLBCL-derived EVs significantly induced apoptosis of T cells (Fig. 8A). Moreover, this EV treatment also decreased IFN-r expression of CD8 + T cells (Fig. 8B), and enabled the increase of immune-regulation molecules, including CTLA-4 (Fig. 8C, D), PD-1, PD-L1, TGF-β, IL-10, and FoxP3 (Fig. 7D). Taken together, the present data further emphasized the immunosuppressive effects of DLBCL-derived EVs on CD8 + T cells.

**Table 1:** The characteristics of canine DLBCL patients

No	Breed	Gender	Age	Stage	Substage
1	Dachshund	SF	10	III	a
2	American Eskimo	CM	9	IV	b
3	Mixed	M	14	III	b
4	Labrador retriever	M	5	V	b
5	Border Collies	SF	8	IV	a
6	Mixed	F	6	IV	b

*M* male, *CM* castrated male, *F* female, *SF* spayed female

## Discussion

The current study demonstrated the immune-modulating effects of canine lymphoma-derived small EVs on primary cultured CD8 + T-cells. Our results showed that small EVs derived from both B cell lymphoma cell line (Fig. 2–5) and clinical samples (Fig. 8) possess similar inhibitory effects on CD8 + T cells. They contributed to decreased cell viability, late apoptosis, and loss of immune activities in CD8 + T-cells. Furthermore, the tumor-derived EVs impaired the CD8 + T-cell functions and induced the formation of regulatory T-cells by enhancing inhibitory cytokines in the tumor microenvironment. Under the influence of EVs, CD8 + T-cell proliferation might be manipulated by the regulation of MAPK cascade, DNA replication, phosphorylation of STAT3, and JAK-STAT signaling pathways. Collectively, our data primarily illuminated the role of lymphoma-derived EVs in immune regulation and propagation in canine CD8 + T-cells.

The regulatory effects of EVs, such as apoptosis, induction of CTLA-4, and reduction of IFN-*r*, were identified in the study. We also found that the DEGs participated in the MAPK and JAK-STAT signaling pathways, consistent with one previous study [62]. The tumor-derived EVs could induce T-cell dysfunction by down-regulating the expression of MAPK and blocking the JAK/STAT pathways, thereby contributing to immune suppression. Notably, in our previous study [2], we identified Fas as an abundant protein in CLBL-1-derived EVs, which possibly predicted the findings shown here. Since Fas, a death receptor ligand that can initiate apoptosis signaling [21], is enriched in EVs, it suggests that EVs have the capability to induce T-cell apoptosis. Previous studies had similar findings, in which the small EVs triggered CD8 + T-cell apoptosis [63], mediated apoptosis-inducing ligands, such as Fas ligand, PD-L1, and TRAIL [11], and induced the cell membrane changes in CD8 + T-cells [12]. These potential mechanisms could explain why the tumor-derived EVs reduce cell viability and induce the cell apoptosis of canine CD8 + T-cells.

EV-targeted tumor therapies have been proposed because they can carry several immunosuppressive molecules, such as CTLA-4, PD-L1, TGF-β1, FasL, and TRAIL, which make them possible candidates for developing targeted therapies [64, 65]. In a recent study, Ding et al. demonstrated that pancreatic cancer-derived EVs carry moR-212-3p, which inhibits the regulatory factor X-associated proteins (RFXAP), an important transcription factor that can lead to immune tolerance [66]. Metastatic melanoma-derived EVs express elevated PD-L1, which facilitates the evasion of immune surveillance by interacting with PD-1, thereby inhibiting anti-tumor responses [67]. Furthermore, EV-derived CTLA-4 promotes proliferation and metastasis in liver cancer [68]. These results showed that tumor-derived EVs could interrupt the immune system and that EV blockade might effectively reverse these immune suppressive behaviors, thereby suppressing tumor development. In the current study, we found that tumor-derived EVs triggered the overexpression of CTLA-4 on the surface of CD8 + T-cells (Fig. 4). This finding echoes that CTLA-4 blockade is a potential method to reverse the immune regulatory abilities resulting from the tumor-derived EVs. In human medicine, several clinical trials using ipilimumab, which blocks the CTLA-4 signal or CTLA-4 inhibitors, have been widely reported and offered prolonged survival with manageable side effects in patients with B-cell lymphoma [69, 70]. Together, the immune-regulating molecules contained within tumor-derived EVs should be seriously considered in cancer treatments.

Regulatory T-cells (Tregs) play a vital role in disturbing immune environments in cancer patients and are widely defined as FoxP3 + CD4 + T-cells [71]. Several molecular mechanisms of Treg-mediated immune regulation have been described, including the suppression mediated by cell–cell contact and the secretion of inhibitory cytokines. Cell–cell contact suppression operates via molecules such as CTLA-4 and LAG-3, which could modulate the immunostimulatory abilities of dendritic cells [72]. Tregs may secret the inhibitory cytokines TGF-β and IL-10, allowing them to out-compete effector cells for the growth factor IL-2, leading to apoptosis of the effector T-cells [73, 74]. Therefore, the Tregs may suppress the activation of antigen-presenting cells and/or inhibit effector T-cell proliferation [71]. Over the past few decades, regulatory CD8 + T-cells (CD8 + Tregs) have gradually been identified, though the phenotypes, functions, and mechanisms that inhibit the immune system remain controversial. Various phenotypes of CD8 + Tregs have been proposed, some examples being CD8 + FoxP3 + [75], CD8 + CD103 + [76], CD8 + CD28- [77], and CD8 + CD122 + [78] T-cells. Similarly, immune suppressive functions have also been reported. The CD8 + Tregs could over-produce IL-10 to suppress the IFN-γ secretion, thus inhibiting CD8 + T-cell proliferation [78]. CD8 + Tregs exert an inhibitory function by cell–cell contact mechanisms through the induction of CTLA-4 expressed on the CD8 + T-cell surface [79]. Moreover, TGF-β has been implicated in the suppressive function of CD8 + Tregs [80]. In the current study, we found that EV-treated CD8 + T-cells shared a highly similar phenotype (Fig. 4), genotype (Fig. 4E and 5A), and even immune behaviors of CD8 + Tregs. First, through inducing the CTLA-4 expression, these cells secreted inhibitory cytokines (IL-10 and TGF-β). Second, EVs elevated the PD-1 and PD-L1 expression in the CD8 + T-cells, thereby triggering cell apoptosis. This finding is in line with the immune behaviors of Tregs, which could directly promote cell apoptosis by induction of PD-1 and PD-L1 in the immune cells. Third, the expression of FoxP3, one of the most representative transcript factors of Tregs, was significantly increased in the CD8 + T-cells after the EV incubation. Collectively, these characteristics of CD8 + T-cells, after incubation with EVs, were highly similar to those of Tregs. To the authors’ best knowledge, the current study is the first to propose that CD8 + Tregs might exist in dogs and to provide marked clues for further investigation.

## Conclusion

This study has reported that lymphoma-derived EVs induce the death and dysfunction of CD8 + T-cells. Furthermore, EVs also trigger the expression of the immune regulatory molecule CTLA-4 on the surface of CD8 + T-cells, and switch the phenotype into regulatory counterparts (CD8 + Tregs). This indicates that EV blockade may be a therapeutic strategy for lymphoma patients. Several immunoregulatory mechanisms were also identified as being responsible for CD8 + T-cell impairment. These include the downregulation of IFN-γ, the production of immunosuppressive cytokines such as IL-10 and TGF-β, and the secretion of angiogenic factors within the pro-tumorigenic microenvironment. Collectively, this study demonstrated the impacts of EVs on the CD8 + T-cells and revealed for the first time the immune behaviors of CD8 + Tregs in dogs. These insights may guide future research on the interaction between lymphoma-derived extracellular vesicles and T-cells.

### Supplementary Information

**Additional file 1: Figure S1.** Characteristics of CLBL-1-derived EVs. Representative figure of the distribution of nanoparticle diameters by Nanosight. The mean, mode, and particle number were analyzed. **Figure S2**. Stimulation and determination of primarily cultured canine CD8+ T cells. (A) PBMCs were collected by the Ficoll-based density gradient and stimulated with IL-2 (2500 IU) and 2-ME (50 µM) from day 0 to 23. The distribution of lymphocytes was defined by FSC and SSC. (B) CD4 and CD8+ T cells were analyzed by flow cytometry (gated on the lymphocyte population). (C) After stimulation, the CD8+ T cells were separated by MACS® separator and the purified CD8+ T cells and the flow through (gated on the distribution of PBMC) were analyzed by flow cytometry. (D) The percentage of purified CD8+ T cells (above 95%) and the flow through (below 5%) were determined. **Figure S3**. Quantification of canine cytokine secretions in the culture media of CD8+ T cells with and with EV incubation. (A) Cytokines released from CD8+ T cells without or (B) with EV incubation (100 µg/mL) were detected using RayBio® C-Series Canine Cytokine Array Kit 1. Cytokines are spotted and those released from the media appear as black dots. Each antibody was spotted in duplicate. (C) The corresponding cytokines were listed. **Table S1**. Description and the PBMC counts of healthy dogs in the study. **Table S2**. Comparisons of CD8+ T cell percentage by various parameters. **Table S3**. Sequences of primers used in quantitative PCR. **Table S4**. The 20 up-regulated genes. **Table S5**. The 19 down-regulated genes.

## Data Availability

The data presented in this study are available on request from the corresponding author.

## Abbreviations

CTLA-4: Cytotoxic T-lymphocyte-associated protein-4
DLBCL: Diffuse large B-cell lymphoma
DEGs: Differentially expressed genes
EVs: Extracellular vesicles
FasL: Fas ligand
HGFR: Hepatocyte growth factor receptor
IL: Interleukin
IFN-r: Interferon-r
PD-1: Programmed cell death-1
PD-L1: Programmed cell death ligand-1
TGF-β: Transforming growth factor-β
VEGF-A: Vascular endothelial growth factor A
