# Supplementary material for: Canine diffuse large B-cell lymphoma downregulates the activity of CD8 + T-cells through tumor-derived extracellular vesicles
Source: Cancer Cell Int. 2023 Oct 26;23:252. doi: 10.1186/s12935-023-03104-4 (PMC10601183; doi:10.1186/s12935-023-03104-4)
Supplement: Supplementary file 1 — Additional file 1: Figure S1. Characteristics of CLBL-1-derived EVs. Representative figure of the distribution of nanoparticle diameters by Nanosight. The mean, mode, and particle number were analyzed. Figure S2. Stimulation and determination of primarily cultured canine CD8+ T cells. (A) PBMCs were collected by the Ficoll-based density gradient and stimulated with IL-2 (2500 IU) and 2-ME (50 µM) from day 0 to 23. The distribution of lymphocytes was defined by FSC and SSC. (B) CD4 and CD8+ T cells were analyzed by flow cytometry (gated on the lymphocyte population). (C) After stimulation, the CD8+ T cells were separated by MACS® separator and the purified CD8+ T cells and the flow through (gated on the distribution of PBMC) were analyzed by flow cytometry. (D) The percentage of purified CD8+ T cells (above 95%) and the flow through (below 5%) were determined. Figure S3. Quantification of canine cytokine secretions in the culture media of CD8+ T cells with and with EV incubation. (A) Cytokines released from CD8+ T cells without or (B) with EV incubation (100 µg/mL) were detected using RayBio® C-Series Canine Cytokine Array Kit 1. Cytokines are spotted and those released from the media appear as black dots. Each antibody was spotted in duplicate. (C) The corresponding cytokines were listed. Table S1. Description and the PBMC counts of healthy dogs in the study. Table S2. Comparisons of CD8+ T cell percentage by various parameters. Table S3. Sequences of primers used in quantitative PCR. Table S4. The 20 up-regulated genes. Table S5. The 19 down-regulated genes. [file 12935_2023_3104_MOESM1_ESM.docx]

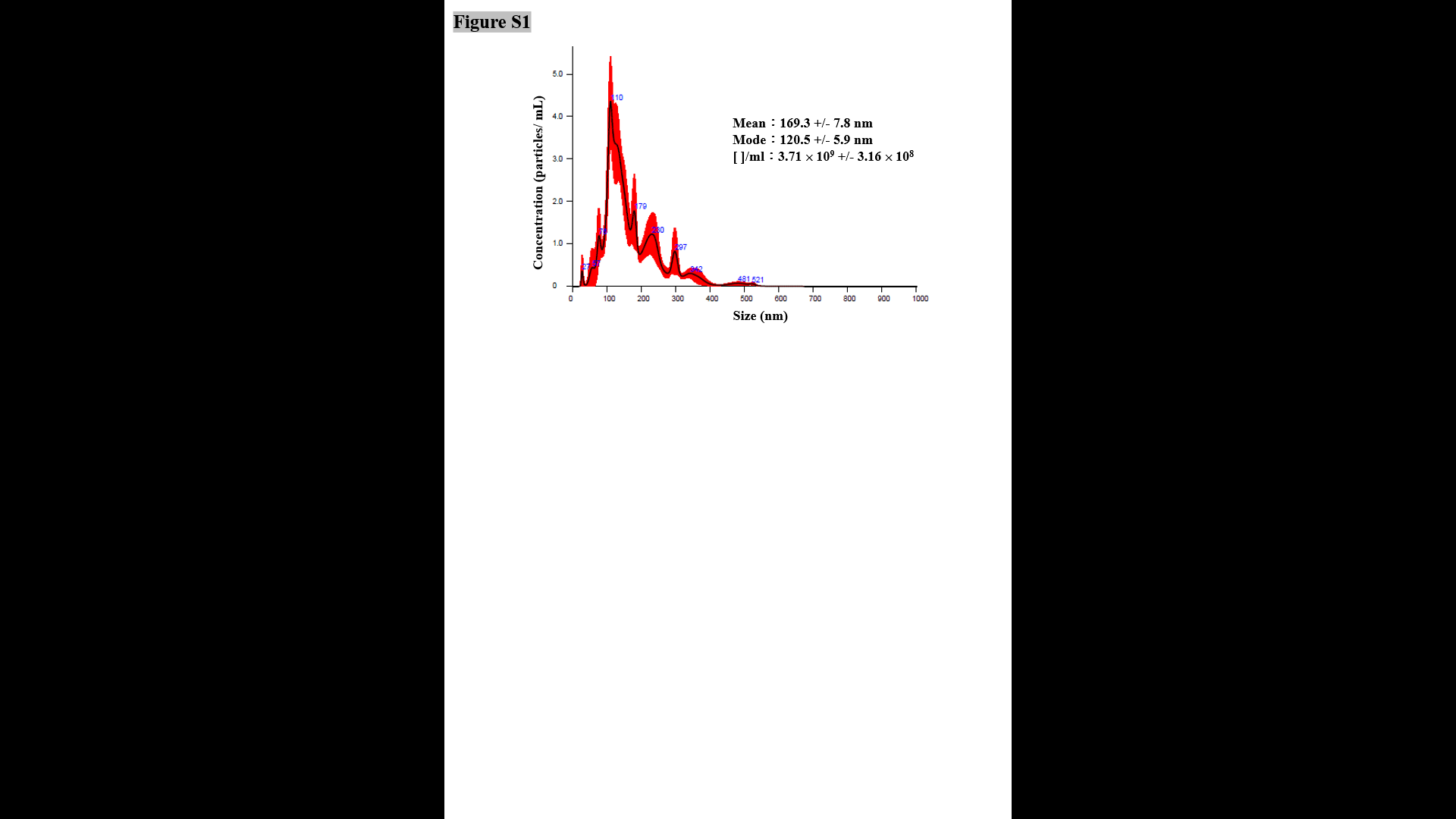


**Figure S1. Characteristics of CLBL-1-derived EVs.** Representative figure of the distribution of nanoparticle diameters by Nanosight. The mean, mode, and particle number were analyzed.


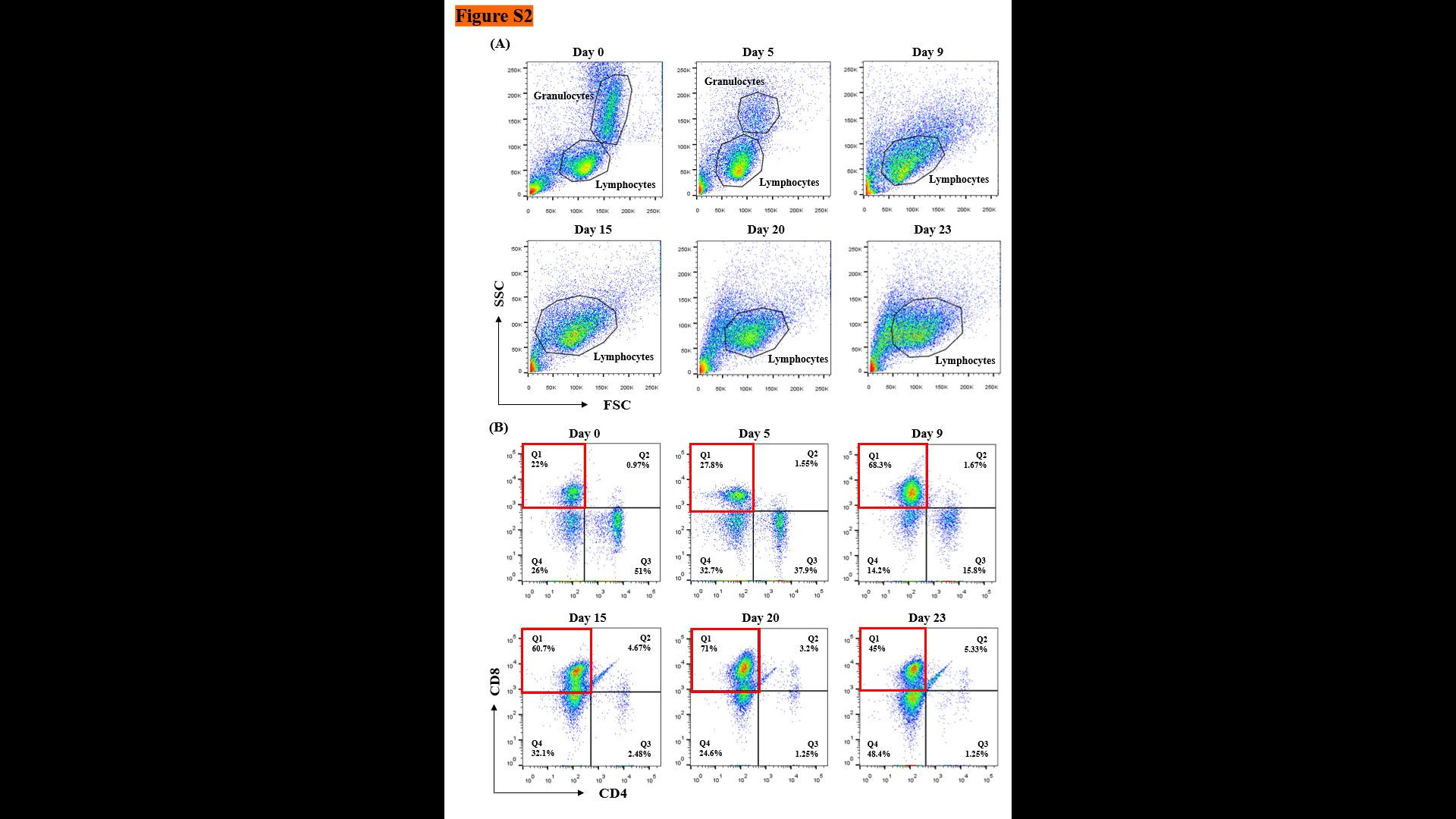


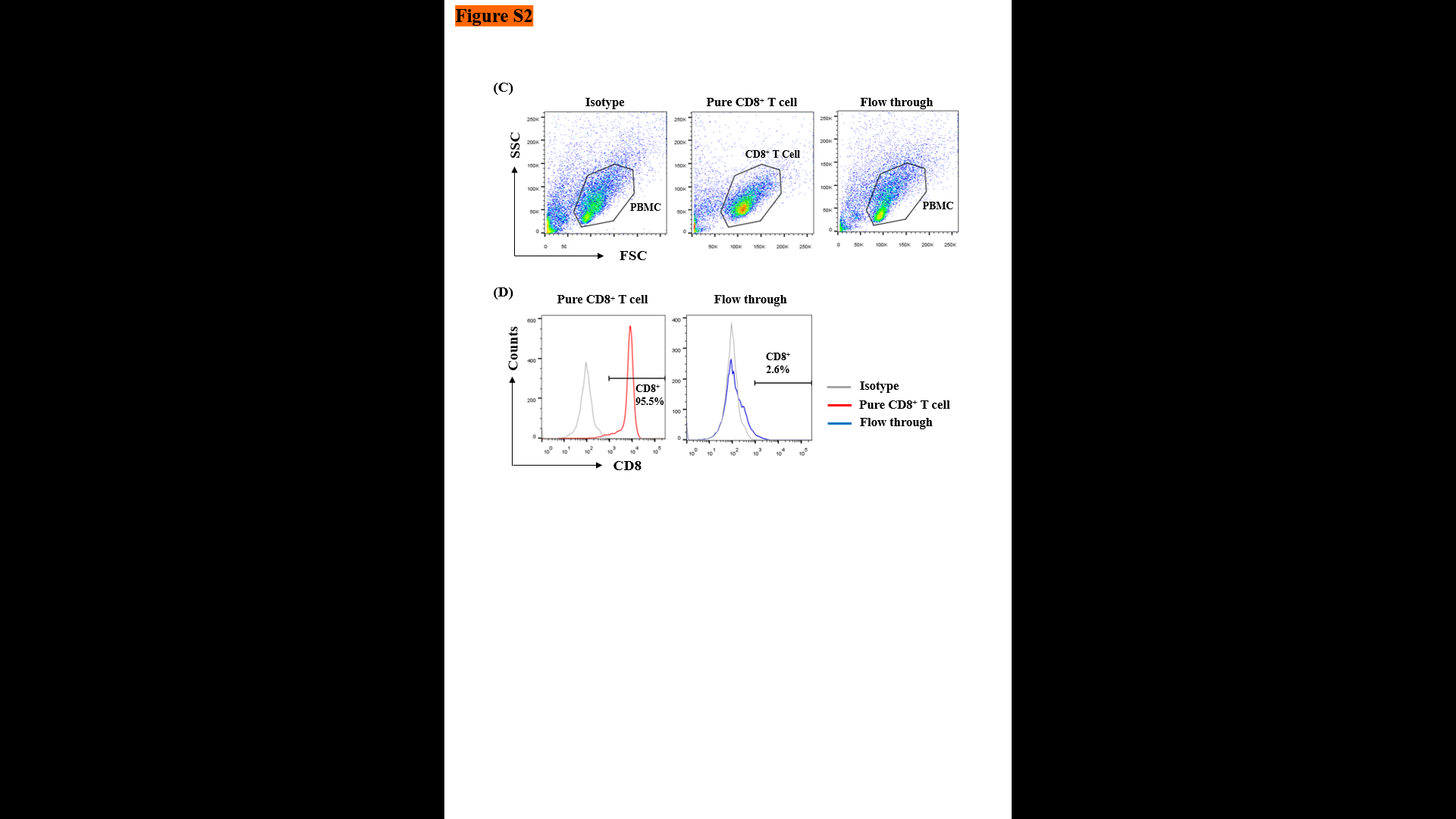


**Figure S2. Stimulation and determination of primarily cultured canine CD8+ T cells.** (A) PBMCs were collected by the Ficoll-based density gradient and stimulated with IL-2 (2500 IU) and 2-ME (50 µM) from day 0 to 23. The distribution of lymphocytes was defined by FSC and SSC. (B) CD4 and CD8+ T cells were analyzed by flow cytometry (gated on the lymphocyte population). (C) After stimulation, the CD8+ T cells were separated by MACS^®^ separator and the purified CD8+ T cells and the flow through (gated on the distribution of PBMC) were analyzed by flow cytometry. (D) The percentage of purified CD8+ T cells (above 95%) and the flow through (below 5%) were determined.


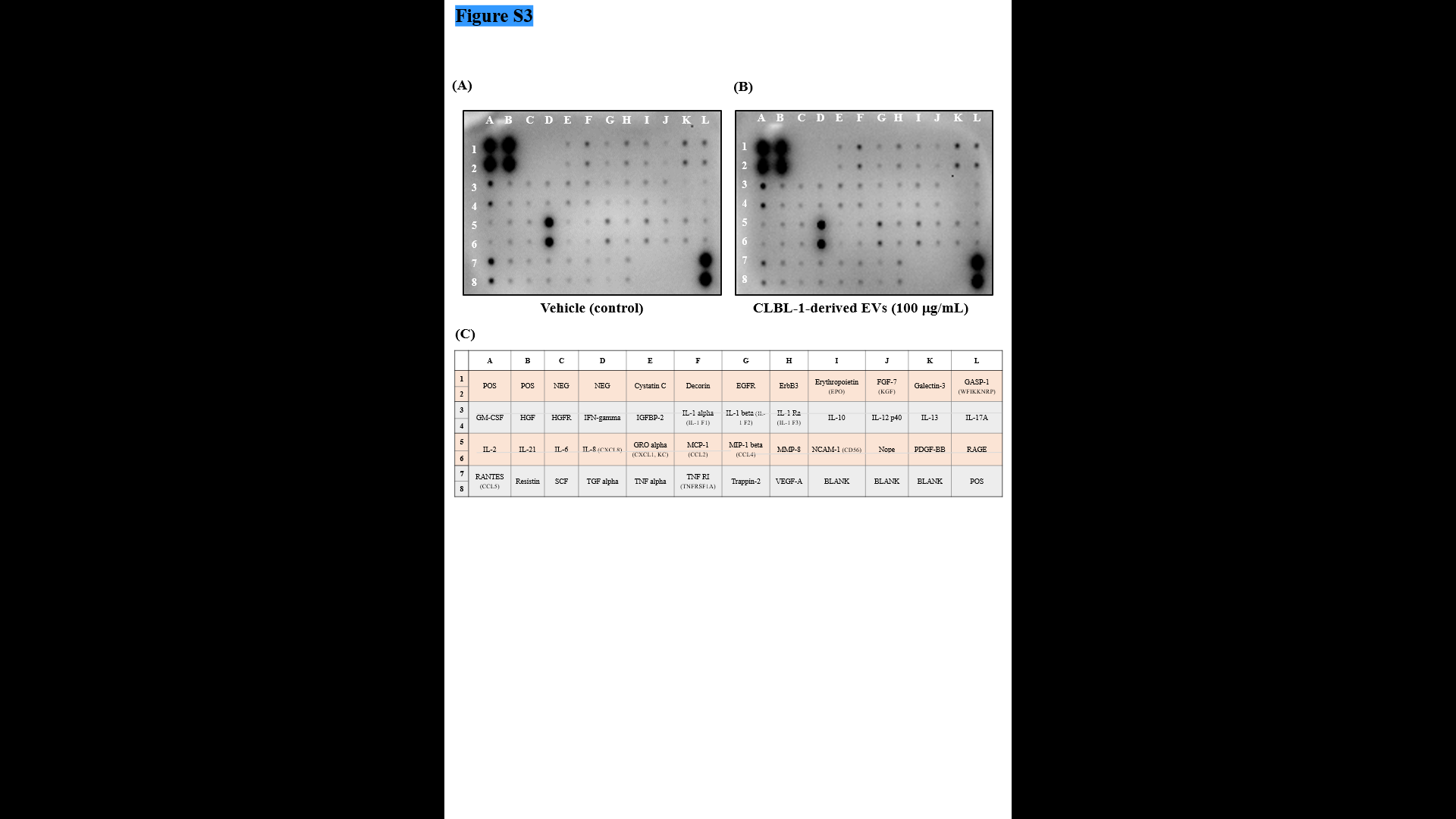


**Figure S3. Quantification of canine cytokine secretions in the culture media of CD8+ T cells with and with EV incubation.** (A) Cytokines released from CD8+ T cells without or (B) with EV incubation (100 µg/mL) were detected using RayBio® C-Series Canine Cytokine Array Kit 1. Cytokines are spotted and those released from the media appear as black dots. Each antibody was spotted in duplicate. (C) The corresponding cytokines were listed.

**Table S1. Description and the PBMC counts of healthy dogs in the study.**

| No. | Gender | Breed | Age (years) | Weight (kg) | PBMC count (10^7^) | CD8+(%); CD4+(%)^1^ |
| --- | --- | --- | --- | --- | --- | --- |
| 1 | F | Pit Bull | 4.0 | 34.0 | 3.89 | 18.9; 46.3 |
| 2 | F | Mixed | 2.0 | 48.0 | 29.5 | 19.8; 55.1 |
| 3 | M | Poodle | 1.2 | 19.0 | 57.6 | 22.7; 38.9 |
| 4 | F | Mixed | 1.0 | 40.0 | 10.5 | 9.1; 23.1 |
| 5 | F | Mixed | 4.0 | 22.0 | 7.38 | 21.2; 49.9 |
| 6 | F | Mixed | 6.0 | 32.0 | 5.37 | 22.1; 52.1 |
| 7 | F | Dogo Argentino | 2.0 | 42.0 | 8.45 | 27.2; 41.7 |
| 8 | M | Mixed | 6.0 | 52.0 | 7.4 | 29.9; 45.1 |
| 9 | F | Mixed | 1.2 | 9.1 | 15.4 | 17.1; 46.0 |
| 10 | F | Mixed | 1.2 | 8.8 | 15,4 | 15.5; 37.2 |
| 11 | M | Mixed | 4.0 | 25.0 | 10.5 | 31.8; 46.8 |
| 12 | F | Mixed | 1.2 | 15.0 | 5.45 | 12.1; 63.4 |
| 13 | M | Greyhound | 1.3 | 30.0 | 14.3 | 18.0; 58.6 |
| 14 | F | Mixed | 1.3 | 11.6 | 16.4 | 17.0; 46.0 |
| 15 | M | Greyhound | 1.0 | 30.0 | 20.0 | 22.6; 42.1 |
| 16 | M | Mixed | 3.0 | 20.9 | 11.3 | 31.2; 37.3 |
| 17 | M | Mixed | 3.0 | 11.0 | 23.3 | 17.9; 55.3 |
| 18 | M | Greyhound | 1.0 | 33.0 | 27.0 | 20.1; 46.0 |
| 19 | F | Mixed | 3.0 | 17.0 | 14.8 | 32.0; 45.0 |
| 20 | F | Dogo Argentino | 1.0 | 30.0 | 20.0 | 28.7; 37.8 |
| 21 | M | Mixed | 4.6 | 20.0 | 12.0 | 14.4; 45.4 |
| 22 | M | Mixed | 2.0 | 52.0 | 40.0 | 26.1; 47.1 |

^1^ Subset of the T cells gated on the lymphocyte population.

**Table S2. Comparisons of CD8+ T cell percentage by various parameters.**

| **Variable** | **CD8+ T cells (%)** | | ***P* value** |
| --- | --- | --- | --- |
|  | N (%) | Average (range) |  |
| **Gender** |  |  | 0.2276 |
| Male | 10 (45.5%) | 23.47 (14.1 - 31.8) |  |
| Female | 12 (54.5%) | 20.05 (9.1 – 32.0) |  |
| **Breed** |  |  | 0.4176 |
| Purebred | 7 (31.8%) | 22.60 (18.0 - 28.7) |  |
| Mixed | 15 (68.2%) | 21.15 (9.1 – 32.0) |  |
| **Age (years)** |  |  | 0.1444 |
| ≦2.5 | 13 (1-2.5) | 19.69 (9.1 - 28.7) |  |
| > 2.5 | 9 (3-6) | 24.38 (14.4 – 32.0) |  |
| **Weight (kg)** |  |  | 0.5619 |
| ≦26 | 11 (8.8-26) | 21.17 (12.1 – 32.0) |  |
| > 26 | 11 (30-52) | 22.05 (9.1 - 29.9) |  |

**Table S3. Sequences of primers used in quantitative PCR.**

| **Gene symbol** | **Primer sequence** | | **Reference** |
| --- | --- | --- | --- |
|  | **Forward sequence** | **Reverse sequence** |  |
| IFN-gamma | GCGCAAGGCGATAAATGAAC | CTGACTCCTTTTCCGCTTCC | NM_001003174 (NCBI) |
| PD-1 | CCCAACACACAGATCAACGAGAG | CAGTAGCAGGACACCCACCA | NM_001314097.1 (NCBI) |
| PD-L1 (CD274) | TGGCAAAACCACCATCACTA | CAGGAAAGGTCCCAGAATCA | NM_001291972 (NCBI) |
| CTLA4 (CD152) | ACTATGTAGGCATGGGAAATGGAAC | AAACAAGCCCGAACTGACTGC | NM_001003106 (NCBI) |
| FoxP3 | AAACAGCACATTCCCAGAGTTC | AGGATGGCCCAGCGGATCAG | [1] |
| TGF-β | AGTTAAAAGCGGAGCAGCATGTGG | GATCCTTGCGGAAGTCAATGTAGAGC | [1] |
| IL-10 | GTCCCTGCTGGAGGACTTTAAGA | TGGTCGGCTCTCCTACATCTCG | [1] |
| OAZ-1 | CTGCTGTAGTAACCTGGGTC | ACATTCAGCCGATTATCAGAGTA | NM_001127234.1 (NCBI) |

**Table S4. The 20 up-regulated genes.**

| No. | Ensembl_gene_id | Symbol | Description | Log2FC_N1 | Pvalue_N1 | Log2FC_N2 | Pvalue_N2 | Log2FC_N3 | Pvalue_N3 |
| --- | --- | --- | --- | --- | --- | --- | --- | --- | --- |
| 1 | ENSCAFG00000008487 | PDE3B | phosphodiesterase 3B | 1.5344 | 4.41E-230 | 0.9171 | 2.71E-57 | 0.7200 | 8.76E-21 |
| 2 | ENSCAFG00000001324 | ATF4 | activating transcription factor 4 | 1.4852 | 2.95E-75 | 1.0055 | 2.93E-38 | 0.8243 | 3.89E-20 |
| 3 | ENSCAFG00000010940 | MCTP2 | multiple C2 and transmembrane  domain containing 2 | 1.8885 | 6.27E-47 | 0.6548 | 7.96E-03 | 0.6931 | 2.39E-02 |
| 4 | ENSCAFG00000029920 | TSC22D3 | TSC22 domain family member 3 | 1.9099 | 2.26E-40 | 1.4237 | 3.36E-12 | 1.0383 | 4.74E-07 |
| 5 | ENSCAFG00000022709 | NA | NA | 1.3582 | 3.41E-39 | 1.6052 | 1.36E-102 | 1.5829 | 9.03E-57 |
| 6 | ENSCAFG00000016034 | TNRC18 | trinucleotide repeat containing 18 | 1.3031 | 1.00E-37 | 0.8288 | 6.29E-07 | 0.7008 | 2.53E-05 |
| 7 | ENSCAFG00000022711 | NA | NA | 0.7316 | 4.53E-18 | 0.6144 | 4.18E-29 | 1.1854 | 6.45E-60 |
| 8 | ENSCAFG00000014827 | EZH1 | enhancer of zeste 1 polycomb  repressive complex 2 subunit | 1.6766 | 4.02E-08 | 1.0917 | 1.51E-04 | 0.8013 | 2.76E-02 |
| 9 | ENSCAFG00000016984 | PER1 | period circadian regulator 1 | 2.5234 | 4.70E-08 | 2.1370 | 3.69E-05 | 1.1173 | 4.74E-02 |
| 10 | ENSCAFG00000011474 | FCMR | Fc fragment of IgM receptor | 1.0671 | 2.78E-07 | 0.9587 | 7.43E-14 | 0.7079 | 9.30E-07 |
| 11 | ENSCAFG00000011850 | KLHL24 | kelch like family member 24 | 1.1579 | 1.11E-06 | 0.9102 | 1.59E-03 | 0.7510 | 2.80E-02 |
| 12 | ENSCAFG00000019565 | CLCN7 | chloride voltage-gated channel 7 | 1.2124 | 4.20E-06 | 1.2702 | 6.24E-07 | 0.6081 | 1.42E-02 |
| 13 | ENSCAFG00000016293 | FAM193B | family with sequence similarity  193 member B | 0.9233 | 6.59E-06 | 1.0678 | 2.66E-09 | 0.6568 | 1.23E-03 |
| 14 | ENSCAFG00000010285 | ABCG1 | ATP binding cassette subfamily  G member 1 | 3.4303 | 2.25E-04 | 1.8352 | 8.48E-03 | 2.5268 | 7.48E-04 |
| 15 | ENSCAFG00000028460 | NA | NA | 2.0683 | 4.35E-04 | 2.9179 | 6.08E-05 | 0.8533 | 3.77E-02 |
| 16 | ENSCAFG00000001424 | PLA2G6 | phospholipase A2 group VI | 0.9177 | 5.39E-04 | 1.4294 | 1.52E-06 | 0.7713 | 1.39E-02 |
| 17 | ENSCAFG00000005589 | CCDC40\|GAA | coiled-coil domain containing 40 | 0.8292 | 1.80E-03 | 1.3866 | 6.95E-09 | 0.9847 | 1.10E-04 |
| 18 | ENSCAFG00000017095 | YPEL3 | yippee like 3 | 0.6549 | 2.25E-03 | 1.1547 | 4.82E-07 | 0.8735 | 3.19E-04 |
| 19 | ENSCAFG00000005048 | IFT172 | intraflagellar transport 172 | 0.9674 | 2.28E-02 | 1.4478 | 7.23E-05 | 0.9431 | 2.03E-02 |
| 20 | ENSCAFG00000022486 | NA | NA | 1.4101 | 2.38E-02 | 1.1707 | 1.75E-03 | 1.2657 | 1.17E-02 |

**Table S5 The 19 down-regulated genes.**

| No. | Ensembl_gene_id | Symbol | Description | Log2FC_N1 | Pvalue_N1 | Log2FC_N2 | Pvalue_N2 | Log2FC_N3 | Pvalue_N3 |
| --- | --- | --- | --- | --- | --- | --- | --- | --- | --- |
| 1 | ENSCAFG00000007344 | MYBL1 | MYB proto-oncogene like 1 | -2.0556 | 3.11E-88 | -1.2880 | 1.35E-22 | -0.6880 | 6.86E-07 |
| 2 | ENSCAFG00000011405 | TXNIP | thioredoxin interacting protein | -1.5707 | 5.32E-42 | -0.9609 | 9.77E-10 | -1.6624 | 7.76E-37 |
| 3 | ENSCAFG00000030292 | LTB | lymphotoxin beta | -2.0813 | 5.85E-28 | -0.7100 | 4.89E-04 | -0.6120 | 1.65E-10 |
| 4 | ENSCAFG00000010293 | CISH | cytokine inducible SH2 containing  protein | -1.3656 | 9.53E-21 | -1.0458 | 2.74E-09 | -0.5958 | 1.67E-07 |
| 5 | ENSCAFG00000004384 | CTSC | cathepsin C | -0.7748 | 3.26E-17 | -1.2383 | 5.10E-23 | -0.6502 | 1.94E-11 |
| 6 | ENSCAFG00000022744 | NA | NA | -1.3969 | 1.62E-09 | -1.7840 | 1.92E-12 | -0.8155 | 2.79E-04 |
| 7 | ENSCAFG00000017539 | LDLR | low density lipoprotein receptor | -2.4401 | 1.89E-08 | -1.1653 | 3.34E-04 | -0.9111 | 9.21E-04 |
| 8 | ENSCAFG00000044650 | IFGGB2 | interferon-inducible GTPase 1-like | -1.1332 | 1.01E-07 | -1.1059 | 6.09E-08 | -0.5876 | 5.06E-04 |
| 9 | ENSCAFG00000030087 | PCLAF | casein kinase 1 gamma 1 | -1.5255 | 1.45E-07 | -0.8037 | 1.39E-04 | -0.6924 | 6.77E-05 |
| 10 | ENSCAFG00000030931 | LIF | LIF interleukin 6 family cytokine | -2.4568 | 1.95E-07 | -1.7980 | 9.09E-06 | -1.0499 | 2.96E-04 |
| 11 | ENSCAFG00000001056 | SQLE | squalene epoxidase | -1.3047 | 4.65E-07 | -1.0826 | 3.31E-09 | -0.6054 | 7.44E-04 |
| 12 | ENSCAFG00000002196 | MCM3 | minichromosome maintenance  complex component 3 | -1.1590 | 2.94E-06 | -0.9415 | 7.58E-07 | -0.6672 | 3.44E-06 |
| 13 | ENSCAFG00000005068 | KCTD12 | potassium channel tetramerization  domain containing 12 | -1.6417 | 5.64E-05 | -1.1661 | 2.20E-02 | -0.8549 | 3.56E-02 |
| 14 | ENSCAFG00000001709 | MCM5 | minichromosome maintenance  complex component 5 | -1.3548 | 1.35E-04 | -1.2484 | 2.28E-07 | -0.7442 | 1.72E-06 |
| 15 | ENSCAFG00000012468 | OSM | oncostatin M | -2.1127 | 1.27E-03 | -1.4744 | 4.58E-02 | -1.0320 | 2.53E-02 |
| 16 | ENSCAFG00000001315 | CTNNA1 | catenin alpha 1 | -0.7855 | 1.74E-03 | -0.8450 | 5.93E-03 | -0.6132 | 2.43E-02 |
| 17 | ENSCAFG00000001204 | KIF20A | kinesin family member 20A | -1.0150 | 1.07E-02 | -1.1974 | 4.89E-04 | -0.7002 | 1.30E-02 |
| 18 | ENSCAFG00000049383 | NA | NA | -4.8800 | 1.28E-02 | -1.4579 | 1.86E-03 | -0.9421 | 4.99E-02 |
| 19 | ENSCAFG00000003174 | DPY19L1 | dpy-19 like C-mannosyltransferase 1 | -1.2748 | 3.14E-02 | -1.7248 | 3.04E-03 | -1.0809 | 2.38E-02 |

**Reference**

1. Biller, B.J.; Elmslie, R.E.; Burnett, R.C.; Avery, A.C.; Dow, S.W. Use of FoxP3 expression to identify regulatory T cells in healthy dogs and dogs with cancer. *Vet Immunol Immunopathol* **2007**, *116*, 69-78, doi:10.1016/j.vetimm.2006.12.002.
